# On the Modeling of Nonlinear Wind-Induced Ice-Drift Ocean Currents at the North Pole

**DOI:** 10.1007/s00021-025-00975-7

**Published:** 2025-10-03

**Authors:** Christian Puntini

**Affiliations:** Faculty of Mathematics, University of Vienna, Oskar-Morgenstern-Platz 1, Vienna, 1090 Austria

**Keywords:** Arctic Ocean, Navier-Stokes equations, Ice-drift current, Asymptotic methods, Ekman spiral, Rotating spherical coordinates, 76M45, 76U60, 35B30, 35C05, 35Q30, 35Q35

## Abstract

Starting from the governing equations for geophysical flows, by means of a thin-shell approximation and a tangent plane approximation, we derive the equations describing, at leading order, the nonlinear ice-drift flow for regions centered around the North Pole. An exact solution is derived in the material/Lagrangian formalism, describing a superposition of oscillations, a mean Ekman flow, and a geostrophic current.

## Introduction

**Fig. 1:**
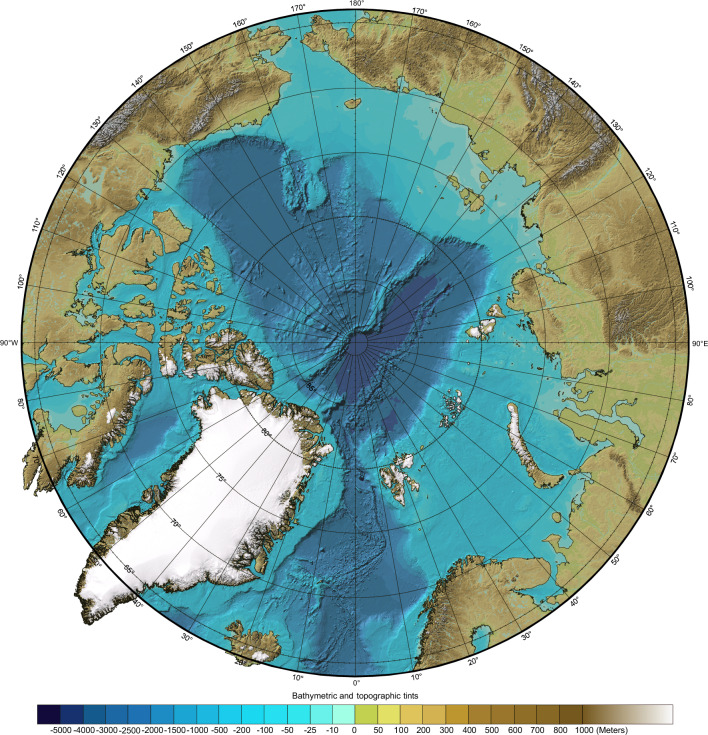
IBCAO bathymetry map of the Arctic Ocean, indicating circles of latitude 5 degrees apart. From https://www.ngdc.noaa.gov/mgg/bathymetry/arctic/currentmap.html. Credit [1]

The Arctic Ocean, centered over the North Pole, is a deep basin with maximum water depths exceeding $4000 \,m$, surrounded by extensive continental shelves with maximum depths between $300 \,m$ and $400 \,m$. This ocean basin connects to the Pacific Ocean via the shallow Bering Strait, with depths of less than $45 \,m$, and to the Atlantic Ocean through the deeper Greenland Sea (maximum depths of $2600 \,m$) and Denmark Strait (maximum depths of $650\,m$). Additionally, shallow channels (with maximum depths under $200\,m$) within the Canadian Arctic and Svalbard archipelagos provide further connections to the Atlantic. See Figure 1.

Atmospheric circulation plays a dominant role in driving the surface circulation of the ocean. Anticyclonic (clockwise) atmospheric flow over the Arctic Ocean basin sets in motion the surface current circulation, represented by the Beaufort Sea Gyre and the Transpolar Drift. These surface currents transport Arctic Water, characterized by temperatures ranging from $-1.8^\circ C$ to $3^\circ C$ and salinities from $31\%$ to $34\%$, across the Arctic Ocean basin. Most of this water is discharged into the North Atlantic Ocean via the East Greenland Current and the Baffin Current.

Sea ice is a dominant feature of polar seas, covering less than $10\%$ of the world’s oceans, with approximately $40\%$ of the world’s sea ice located within the Arctic Ocean basin.

The presence of sea ice in the Arctic Ocean and its marginal seas, including Baffin Bay, Hudson Bay, and the Barent Sea has several important effects on the physical oceanography of the region: the surface water temperature in this region remains near the freezing point dictated by its salinity, while the formation of sea ice expels salt in a process known as brine rejection, which increases surface water density and drives the thermohaline circulation. Additionally, winds transfer momentum from the atmosphere to the ocean surface through the sea ice cover, while the seasonal variable albedo of sea ice modulates the absorption and reflection of sunlight, affecting the energy exchange at the ocean surface and influencing the melting of the sea ice.

**Fig. 2:**
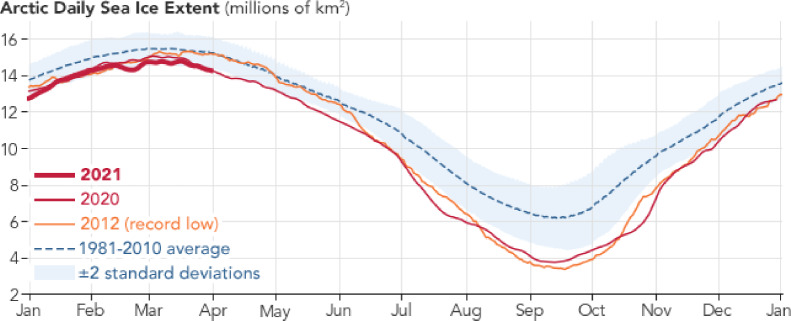
Arctic daily sea ice extent (millions of $km^2$), up to April 2021. From NASA’s Earth Observatory https://earthobservatory.nasa.gov/world-of-change/sea-ice-arctic

The northernmost part of the Arctic Ocean is entirely covered by a thin layer of sea ice, about $2\, m$ thick during the winter, with summer sea-ice extent typically around one third of the winter extent. The winter maximum occurs in March, while the sea-ice minimum is in September (see [2] and Figure 2).

**Fig. 3:**
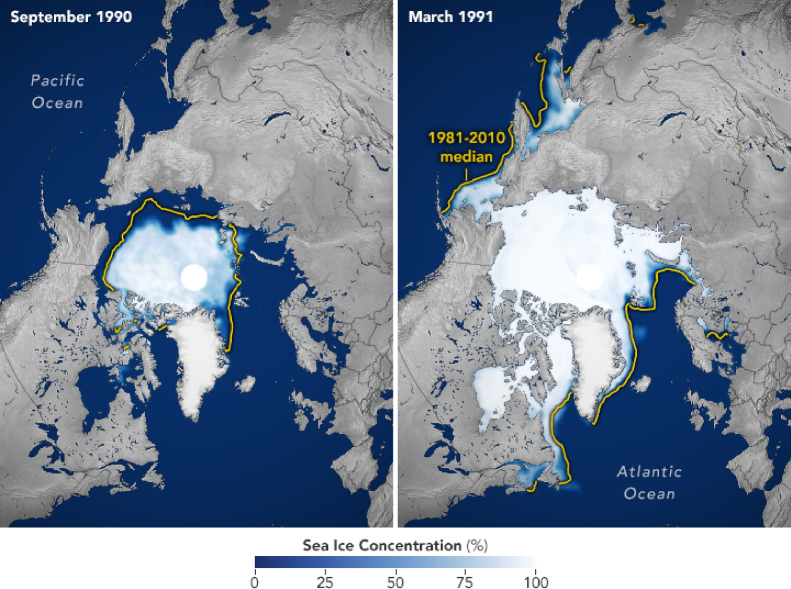
Average concentration of sea ice for the first available annual maxima and minima. From NASA’s Earth Observatory https://earthobservatory.nasa.gov/world-of-change/sea-ice-arctic

**Fig. 4:**
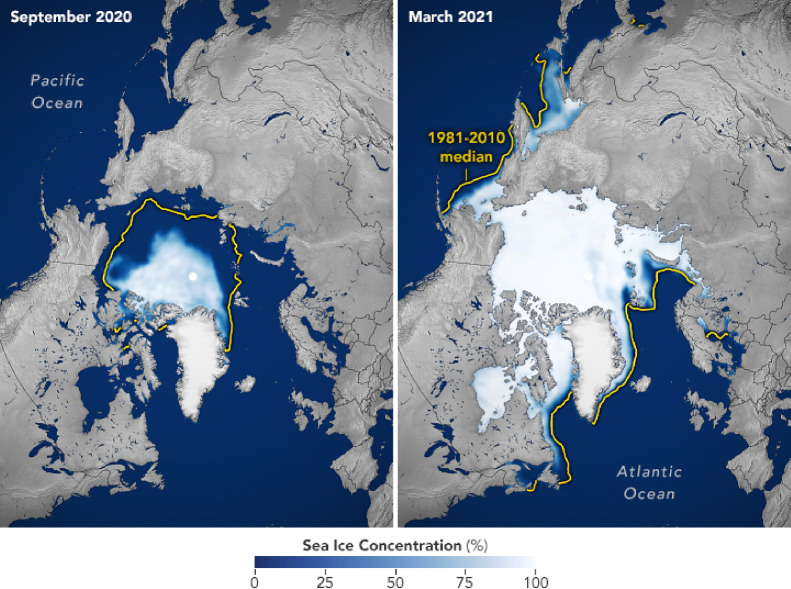
Average concentration of sea ice for the latest available annual maxima and minima. From NASA’s Earth Observatory https://earthobservatory.nasa.gov/world-of-change/sea-ice-arctic

Since the start of the satellite record in 1979, a linear trend indicates that summer (September) sea-ice surface has been declining at a rate of about 1 million $km^2$ per decade, with recent years seeing only about 4.5 million $km^2$ of sea ice in September. See Figures 3, 4. This loss in sea-ice area is accompanied by a decrease in sea-ice volume, with a shift to thinner, more mobile sea-ice packs. In the 1980s, average winter [fall] sea-ice thickness was around $3.6\, m$ [$2.7\,m$], while in 2018 it had decreased to about $2\,m$ [$1.5\,m$]. The amount and mobility of sea ice play a significant role in driving large-scale ocean circulation, as sea ice acts as a critical mediator of wind stress in the Arctic. In addition, changes in Arctic Ocean conditions, such as warming, freshening, and shifts in stratification, circulation dynamics, and momentum transfer to the ocean are closely linked to these sea-ice changes (see [2] and the references therein).

Given the evident importance of the Arctic Ocean, it is therefore crucial to understand the variety of phenomena characterizing this basin. The difficulty of physical observations (due to harsh meteorological conditions and to the presence of sea-ice) makes the theoretical approach based on the careful (asymptotic) analysis of the governing equations of fluid mechanics particularly important (and suitable) (see [3] for a survey on this method). Such an approach was recently pursued for the study of the dynamics of the Beaufort Gyre (see e.g. [4]) and of the Transpolar Drift Current (see e.g. [4, 5] or [6]).

In this article, we study, adopting this aforementioned approach, the nonlinear flows under the sea-ice cover induced by wind (which exerts a stress on the ice sheet, in turn inducing a reduced stress on the water surface), extending the work in [7] and [8], which was limited to regions outside the Amundsen Basin (where the North Pole is located, thus avoiding the singularity issue of the convergence of the meridians at the pole linked to the use of classical spherical coordinates). To avoid this inconvenience, we will adopt the rotated spherical coordinates (having the singularity at the Equator) developed in [4] and [9].

At latitudes away from the equator, the oceanic flow below a depth of about $100\,m$ typically features a geostrophic balance. However, near the surface, wind-driven turbulence transfers momentum into the ocean, generating currents that overlay the underlying geostrophic flow. The resulting force balance in these currents involves the Coriolis force and the frictional forces induced by the wind. This region, where these frictional effects are significant, is known as the Ekman layer, named after V. W. Ekman, who first developed a mathematical model, in 1905 [10], to describe the behavior of wind-driven surface currents in order to explain the observations made by F. Nansen during the 1893-1896 Arctic expedition on board of the Fram vessel that sea ice drifts somewhat to the right of the prevailing wind direction (see [11] for a summary of Ekman’s work).

Ekman’s explicit solution is applicable to a uniform, steady wind blowing over a homogeneous ocean with a constant eddy viscosity. In this scenario: the surface flow is oriented at a $45^{\circ }$ angle to the wind, with the direction being to the right in the Northern Hemisphere and to the left in the Southern Hemisphere, and as one dives deeper into the water, the current speed gradually decreases, and the direction of flow rotates further away from the wind, following a spiral pattern. On average, the wind-driven motion, known as Ekman transport, occurs at a right angle to the wind direction, to the right in the Northern Hemisphere and to the left in the Southern Hemisphere. These conclusions hold for cases with depth-dependent eddy viscosity (see [12]), though the first point only qualitatively matches observations, with deflection angles observed in the range of $10^{\circ }$ to $75^{\circ }$ (see [13] and the references therein). The discrepancy between the predicted and observed deflection angles can be mostly attributed to the assumption of constant vertical eddy viscosity in the original model (see [13] for a three-valued piecewise-constant eddy viscosity, or [14] for depth and time dependent eddy viscosity).

As eddy viscosity is just a simple model to “close” the turbulence problem, based on the hypothesis that Reynolds stresses are proportional to the mean velocity gradient and representing no physical characteristic of the fluid (but being function of flow conditions), there were numerous attempts to improve the Ekman solution by choosing a better turbulence model (see [15]). We work with the assumption of constant eddy viscosity. The exploration of more intricate models will be the object of subsequent investigations.

Extending [7] and [8], we provide an exact description of the nonlinear flow, which consists of oscillations superimposed on an Ekman mean spiraling current. However, in our case, the stress on the ocean surface is not directly generated by the wind but rather by the ice motion, whereas in [7] and [8], the stress is induced by the wind, ice, or a combination of both. Differently from [7] and [8] our solution also features a background geostrophic flow, due to the presence of the Transpolar Drift Current.

The paper is structured as follows: in Section 2, after recalling the set of rotated spherical coordinates developed in [4] suitable for the analysis of fluid flows in regions centered around the poles (where classical spherical coordinates fail) we derive the governing equations of our interest, starting from the Navier-Stokes and continuity equations via a thin-shell approximation coupled with a tangent plane approximation. We also describe the boundary conditions associated with the presence of ice and its interaction with the water beneath it.

In Section 3 we present the solution for the ice-drift nonlinear flow, obtained via a Lagrangian formulation of the flow variables. Such a solution features oscillations superimposed on an Ekman-type mean spiraling current and on the background geostrophic component of the Transpolar Drift Current. Moreover, it will be shown that the boundary conditions uniquely determine the surface current, therefore explaining how, given accurate field data about the ice motion, the mean surface current is connected to the ice-sea stress.

Finally, we conclude with a discussion of the results in Section 4.

In Appendix A we derive the Navier-Stokes and continuity equations in their most general form in classical spherical coordinates (A.2.1) and rotated spherical coordinates (A.2.2), suitable for the study of the geophysical fluid dynamics of oceans and of the atmosphere. Only two assumptions will be made: the fluid is Newtonian and the viscosity varies only with depth. The scope is also to correct some errors present in the literature, see e.g. [4] or [9]. The derivation is preceded by a brief review basics of differential geometry, with a focus on orthogonal curvilinear coordinate systems (A.1), in order to provide explicit expressions of the differential operators used in the subsequent derivation of the equations of motion.

## Governing Equations

We assume the Earth to be a sphere of radius $R'\approx 6371\ km$, disregarding the exact form of Earth’s geoid, though appropriate adjustments to its shape can be incorporated (see [16]), and define a Cartesian coordinate system $({\textbf{e}}_1^{\dag }, {\textbf{e}}_2^{\dag }, {\textbf{e}}_3^{\dag })$ positioned at the center of the Earth, with ${\textbf{e}}_1^{\dag }$ pointing towards the North Pole, ${\textbf{e}}_2^{\dag }$ pointing towards Null Island and ${\textbf{e}}_3^{\dag }$ pointing to East. This “new” Cartesian coordinate system can be thought of as a cyclical permutation of the classical Cartesian coordinate system at the Earth’s center and on which classical spherical coordinates are based. See Appendix A for a detailed description of both the classical and this rotated spherical coordinate system, and their relation.

Consider the set of the associated, right-handed, spherical coordinates $(\varphi ^{\dag }, \theta ^{\dag }, r')$, where $\theta ^{\dag }\in [-\frac{\pi }{2},\frac{\pi }{2}]$ and $\varphi ^{\dag }\in [0,2\pi )$ are the azimuthal and meridional angles, respectively, and $r'$ is the distance from Earth’s center. The coordinates of a point *P* on the Earth are, with respect to the coordinates $(\varphi ^{\dag },\theta ^{\dag },r')$,

1 $$\begin{aligned} r'\cos \theta ^{\dag }\cos \varphi ^{\dag }{\textbf{e}}_{1}^{\dag }+r'\cos \theta ^{\dag }\sin \varphi ^{\dag }{\textbf{e}}_{2}^{\dag }+r'\sin \theta ^{\dag }{\textbf{e}}_{3}^{\dag }, \end{aligned}$$

and the North Pole has coordinates $\varphi ^{\dag }=\pi /2, \theta ^{\dag }=0, r'=R'$. Associated with the unit vectors $({\textbf{e}}_{\varphi }^{\dag }, {\textbf{e}}_{\theta }^{\dag }, {\textbf{e}}_r)$ in this $(\varphi ^{\dag }, \theta ^{\dag }, r')$-system are the velocity components $(u'_{\dag }, v'_{\dag }, w'_{\dag })$: $u'_{\dag }$ points from East to West, $v'_{\dag }$ from North to South and $w'_{\dag }$ points upward.

We use primes to represent dimensional (physical) variables. The prime will be removed once we introduce the appropriate set of non-dimensional variables.

In the new rotated spherical coordinates the momentum equations are

2 $$\begin{aligned} \begin{aligned}&\rho ' \frac{D}{D t'}\begin{pmatrix} u'_{\dag } \\ v'_{\dag }\\ w'_{\dag }\\ \end{pmatrix}+\frac{\rho '}{r'}\begin{pmatrix}-u'_{\dag }v'_{\dag }\tan \theta ^{\dag } + u'_{\dag }w'_{\dag }\\ u'^2_{\dag }\tan \theta ^{\dag }+v'_{\dag }w'_{\dag }\\ -u'^2_{\dag }-v'^2_{\dag } \end{pmatrix}\\&\quad +2\rho '\Omega '\begin{pmatrix} -v'_{\dag }\sin \varphi ^{\dag }\cos \theta ^{\dag }-w'_{\dag }\sin \varphi ^{\dag }\sin \theta ^{\dag }\\ u'_{\dag }\sin \varphi ^{\dag }\cos \theta ^{\dag }-w'_{\dag }\cos \varphi ^{\dag }\\ u'_{\dag } \sin \varphi ^{\dag }\sin \theta ^{\dag } +v'_{\dag } \cos \varphi ^{\dag } \end{pmatrix}\\&\quad +\rho ' r' \Omega '^2\begin{pmatrix} \sin \varphi ^{\dag }\cos \varphi ^{\dag }\cos \theta ^{\dag }\\ -\sin ^2\varphi ^{\dag } \sin \theta ^{\dag }\cos \theta ^{\dag }\\ -\cos ^2\varphi ^{\dag }\cos ^2\theta ^{\dag } -\sin ^2\theta ^{\dag } \end{pmatrix} \\&=-\nabla 'p' +\rho \begin{pmatrix} 0\\ 0\\ -g'\frac{R'^2}{ r'^2}\\ \end{pmatrix} +\Delta '_{\mu } \begin{pmatrix} u'_{\dag }\\ v'_{\dag }\\ w'_{\dag } \end{pmatrix}-\frac{1}{3}\begin{pmatrix} \frac{1}{r'\cos \theta ^{\dag }}\frac{\partial }{\partial \varphi ^{\dag }}\left( \frac{\mu '_H}{\rho '} \frac{D\rho '}{D t'}\right) \\ \frac{1}{r'}\frac{\partial }{\partial \theta ^{\dag }}\left( \frac{\mu '_H}{\rho '} \frac{D\rho '}{D t'}\right) \\ \frac{\partial }{\partial r'}\left( \frac{\mu '_V}{\rho '} \frac{D\rho '}{D t'}\right) \end{pmatrix}\\&\quad -\frac{1}{r'^2\cos ^2\theta ^{\dag }}\begin{pmatrix} \mu '_H u'_{\dag }\\ \mu '_H v'_{\dag }\\ 2\mu '_V(w'_{\dag }\cos ^2\theta ^{\dag }-v'_{\dag }\sin \theta ^{\dag }\cos \theta ^{\dag }) \end{pmatrix}+\frac{2\mu '_H}{r'^2}\frac{\partial }{\partial \theta ^{\dag }}\begin{pmatrix} 0\\ w'_{\dag }\\ -v'_{\dag } \end{pmatrix}\\&\quad + \frac{2\mu '_H}{r'^2\cos \theta ^{\dag }}\frac{\partial }{\partial \varphi ^{\dag }}\begin{pmatrix} w'_{\dag }-v'_{\dag }\tan \theta ^{\dag }\\ u'_{\dag }\tan \theta ^{\dag }\\ -u'_{\dag } \end{pmatrix}+ \frac{d \mu '_V}{dr'}r' \begin{pmatrix} \frac{\partial }{\partial r'}\left( \frac{u'_{\dag }}{r'}\right) \\ \frac{\partial }{\partial r'}\left( \frac{v'_{\dag }}{r'}\right) \\ {0} \end{pmatrix}\\&\quad +\frac{d \mu '_H}{d r'} \begin{pmatrix} \frac{1}{r'\cos \theta }\frac{\partial {w'_{\dag }}}{\partial \varphi ^{\dag }}\\ \frac{1}{r'} \frac{\partial {w'_{\dag }}}{\partial \theta ^{\dag }}\\ {0} \end{pmatrix}+ \frac{d \mu '_V}{dr'} \begin{pmatrix} 0\\ 0\\ 2 \frac{\partial w'_{\dag }}{\partial r'} \end{pmatrix}\\&\quad +\frac{d \mu '_V}{dr'}\begin{pmatrix} 0\\ 0\\ \frac{1}{r' \cos \theta ^{\dag }} \frac{\partial u'_{\dag }}{\partial \varphi ^{\dag }} + \frac{1}{r' \cos \theta ^{\dag }} \frac{\partial }{\partial \theta ^{\dag }} \left( v'_{\dag } \cos \theta ^{\dag } \right) + \frac{1}{r'^2} \frac{\partial }{\partial r'} \left( r'^2 w'_{\dag } \right) \end{pmatrix}, \end{aligned} \end{aligned}$$

where

3 $$\begin{aligned} \begin{aligned} \nabla '&= \left( \frac{1}{r'\cos \theta ^{\dag }}\frac{\partial }{\partial \varphi ^{\dag }},\ \frac{1}{r'}\frac{\partial }{\partial \theta ^{\dag }} ,\ \frac{\partial }{\partial r'} \right) ,\\ \frac{D}{Dt'}&=\frac{\partial }{\partial t'}+ \frac{u'_{\dag }}{r' \cos \theta ^{\dag }} \frac{\partial }{\partial \varphi ^{\dag }} + \frac{v'_{\dag }}{r'} \frac{\partial }{\partial \theta ^{\dag }} + w'_{\dag }\frac{\partial }{\partial r'}, \\ \Delta '_{\mu }&=\mu '_V \left( \frac{\partial ^2}{\partial r'^2}+ \frac{2}{r'}\frac{\partial }{\partial r'}\right) + \frac{\mu '_H}{r'^2}\left( \frac{1}{ \cos ^2 \theta ^{\dag }} \frac{\partial ^2 }{\partial \varphi ^{\dag 2}}+\frac{\partial ^2}{\partial \theta ^{\dag 2}} - \tan \theta ^{\dag }\frac{\partial }{\partial \theta ^{\dag }}\right) , \end{aligned} \end{aligned}$$

while the continuity equation is

4 $$\begin{aligned} \frac{D'\rho }{Dt'}+\rho '\left[ \frac{1}{r' \cos \theta ^{\dag }} \frac{\partial u'_{\dag }}{\partial \varphi ^{\dag }} + \frac{1}{r' \cos \theta ^{\dag }} \frac{\partial }{\partial \theta ^{\dag }} \left( v'_{\dag } \cos \theta ^{\dag } \right) + \frac{1}{r'^2} \frac{\partial }{\partial r'} \left( r'^2 w'_{\dag }\right) \right] =0; \end{aligned}$$

$R' \approx 6371\ km$ is the Earth’s radius, $g' \approx 9.81 \ m\, s^{-2}$ is the average acceleration of gravity at the surface of the Earth, $\rho '$ is the fluid density, $\mu '_V$ and $\mu '_H$ are the vertical and horizontal dynamic viscosities and $\Omega ' \approx 7.29\cdot 10^{-5}\ s^{-1}$, is the angular velocity of Earth’s rotation around the ${\textbf{e}}_{1}^{\dag }$-axis.

Moreover, we remark that even if the equation of state of seawater (derived from the first law of thermodynamics) is determined empirically in practice (see [17]), and shows a complex relationship between density, temperature, and salinity, particularly in relatively shallow layers (see [18]), under adiabatic conditions and assuming near-conservation of salinity, it simplifies to the equation of mass conservation (see [19]):

5 $$\begin{aligned} \frac{D\rho '}{D t'}=0. \end{aligned}$$

Although the thermohaline structure of the Arctic Ocean is intricate, the large excess of freshwater (resulting from direct precipitation and significant river runoff) forms a low-density surface layer that significantly reduces heat flux (see [20])^1^. Since changes in salinity in the upper Arctic Ocean (up to about $5\%$) are generally seasonal (see [23]), and thus negligible over the short time scale of days, we can adopt the mass conservation condition from equation (5).

Moreover, assuming that the density of the fluid varies only with depth, namely $\rho '=\rho '(z)$, and that the dynamic viscosities are constant, namely $\frac{ d \mu '_V}{dr}=\frac{ d \mu '_H}{dr}=0$, we have that (2) reduces to (up to dividing by $\rho '$):

6 $$\begin{aligned} \begin{aligned}&\frac{D}{D t'}\begin{pmatrix} u'_{\dag } \\ v'_{\dag }\\ w'_{\dag }\\ \end{pmatrix}+\frac{1}{r'}\begin{pmatrix}-u'_{\dag }v'_{\dag }\tan \theta ^{\dag } + u'_{\dag }w'_{\dag }\\ u'^2_{\dag }\tan \theta ^{\dag }+v'_{\dag }w'_{\dag }\\ -u'^2_{\dag }-v'^2_{\dag } \end{pmatrix}\\&\quad +2\Omega '\begin{pmatrix} -v'_{\dag }\sin \varphi ^{\dag }\cos \theta ^{\dag }-w'_{\dag }\sin \varphi ^{\dag }\sin \theta ^{\dag }\\ u'_{\dag }\sin \varphi ^{\dag }\cos \theta ^{\dag }-w'_{\dag }\cos \varphi ^{\dag }\\ u'_{\dag } \sin \varphi ^{\dag }\sin \theta ^{\dag } +v'_{\dag } \cos \varphi ^{\dag } \end{pmatrix}\\&\quad +r' \Omega '^2\begin{pmatrix} \sin \varphi ^{\dag }\cos \varphi ^{\dag }\cos \theta ^{\dag }\\ -\sin ^2\varphi ^{\dag } \sin \theta ^{\dag }\cos \theta ^{\dag }\\ -\cos ^2\varphi ^{\dag }\cos ^2\theta ^{\dag } -\sin ^2\theta ^{\dag } \end{pmatrix} = -\frac{1}{\rho '}\nabla 'p' +\begin{pmatrix} 0\\ 0\\ -g'\frac{R'^2}{ r'^2}\\ \end{pmatrix}\\&\quad +\frac{1}{\rho '}\Delta '_{\mu } \begin{pmatrix} u'_{\dag }\\ v'_{\dag }\\ w'_{\dag } \end{pmatrix} -\frac{1}{r'^2\cos ^2\theta ^{\dag }}\begin{pmatrix} A'_H u'_{\dag }\\ A'_H v'_{\dag }\\ 2A'_V(w'_{\dag }\cos ^2\theta ^{\dag }-v'_{\dag }\sin \theta ^{\dag }\cos \theta ^{\dag }) \end{pmatrix}\\&\quad +\frac{2A'_H}{r'^2}\frac{\partial }{\partial \theta ^{\dag }}\begin{pmatrix} 0\\ w'_{\dag }\\ -v'_{\dag } \end{pmatrix}+ \frac{2A'_H}{r'^2\cos \theta ^{\dag }}\frac{\partial }{\partial \varphi ^{\dag }}\begin{pmatrix} w'_{\dag }-v'_{\dag }\tan \theta ^{\dag }\\ u'_{\dag }\tan \theta ^{\dag }\\ -u'_{\dag } \end{pmatrix}, \end{aligned} \end{aligned}$$

where

7 $$\begin{aligned} A'_H(z)=\frac{\mu '_H}{\rho '(z)}\qquad \text {and}\qquad A'_V(z)=\frac{\mu '_V}{\rho '(z)} \end{aligned}$$

are the horizontal and vertical kinematic eddy viscosities, while the continuity equation (4) reduces to

8 $$\begin{aligned} \frac{1}{r' \cos \theta ^{\dag }} \frac{\partial u'_{\dag }}{\partial \varphi ^{\dag }} + \frac{1}{r' \cos \theta ^{\dag }} \frac{\partial }{\partial \theta ^{\dag }} \left( v'_{\dag } \cos \theta ^{\dag } \right) + \frac{1}{r'^2} \frac{\partial }{\partial r} \left( r'^2 w ^{\dag }\right) =0. \end{aligned}$$

### The Governing Equations at Leading Order

Asymptotic expansions allow for the extraction of the main structure of the governing equations. To perform them, it is necessary to first non-dimensionalize the governing equations using physical scales that are representative of the specific phenomena, thereby clarifying the relative magnitudes of the terms by introducing suitable parameters that facilitate the development of asymptotic expansions. This approach makes it mathematically possible to obtain order-of-magnitude estimates for the relative importance of various factors. To apply this method to arctic ice-drift currents, we define the relevant physical scales (see [7] and the references therein):

9 $$\begin{aligned} \begin{aligned}&R'\approx 6370\ km\qquad&\text {Earth radius,}\\&H'\approx 50\ m\qquad&\text {mean depth of the arctic sub-surface layer,}\\&L'\approx 10\ km&\text {horizontal length scale,}\\&\bar{\rho '}\approx 1030\ kg\, m^{-3}&\text {average density of the arctic ocean,}\\&U' \approx 0.1\ m\, s^{-1}&\text {horizontal velocity scale.} \end{aligned} \end{aligned}$$

The non-dimensional variables $t, z, u, v, w, \rho , p, A_H, A_V$ are defined by

10 $$\begin{aligned} \begin{aligned}&t'=\frac{L'}{U'}t,\qquad p'=p'_{atm}+\bar{\rho '}U'^2 p+\bar{\rho '}g'H'\int _z^0\rho (s)ds,\\&r'=R'+H' z=R'(1+\epsilon z),\qquad (u'_{\dag }, v'_{\dag }, w'_{\dag })=U'(u,v,\kappa w),\\&\rho '=\bar{\rho '}\rho , \qquad A'_H=U'H' A_H, \qquad A'_V=\frac{U'H'^2}{L'}A_V, \end{aligned} \end{aligned}$$

while the horizontal spatial variables *x*, *y* will be defined later using a tangent plane approximation. The scaling (10) gives $t'\approx 1\, \text {day}$, $A'_H\approx 5\, m^2\, s^{-1}$ and $A'_V\approx 0.025\, m^2\, s^{-1}$. Moreover, we introduce two adimensional parameters

11 $$\begin{aligned} \begin{aligned}&\epsilon =\frac{H'}{R'}\approx 10^{-5}\qquad \text {thin-shell parameter},\\&\delta =\frac{H'}{L'}\approx 5\cdot 10^{-3}\qquad \text {aspect-ratio parameter}, \end{aligned} \end{aligned}$$

and, as vertical velocities are of the order of approximately $10^{-6}\ m\, s^{-1}$, while for the horizontal velocity scale $U' \approx 0.1\ m\, s^{-1}$ (see [7]), we get that, for consistency, $\kappa ={\mathcal {O}}(\epsilon )$ (see [4, 6]). We choose

12 $$\begin{aligned} \kappa =\epsilon \end{aligned}$$

and lastly we define

13 $$\begin{aligned} f= \frac{2\Omega ' L'}{U'}\approx 14.5, \end{aligned}$$

which is the inverse of the Rossby number $Ro=\nicefrac {U'}{2\Omega 'L'}$. Using the previous scaling, (6) became

14 $$\begin{aligned} \begin{aligned}&\left[ \frac{1}{L'}\frac{\partial }{\partial t}+ \frac{u}{R'(1+\epsilon z) \cos \theta ^{\dag }} \frac{\partial }{\partial \varphi ^{\dag }} + \frac{v}{R'(1+\epsilon z) } \frac{\partial }{\partial \theta ^{\dag }} + \frac{\epsilon w}{H'}\frac{\partial }{\partial z} \right] \begin{pmatrix} u \\ v\\ \epsilon w\\ \end{pmatrix}U'^2\\&\quad +\frac{U'^2}{R'(1+\epsilon z)}\begin{pmatrix}-uv\tan \theta ^{\dag } + \epsilon uw\\ u^2\tan \theta ^{\dag }+\epsilon vw\\ -u^2-v^2 \end{pmatrix}\\&\quad +\frac{2\Omega '}{U'}U'^2\begin{pmatrix} -v\sin \varphi ^{\dag }\cos \theta ^{\dag }-\epsilon w\sin \varphi ^{\dag }\sin \theta ^{\dag }\\ u \sin \varphi ^{\dag }\cos \theta ^{\dag }-\epsilon w \cos \varphi ^{\dag }\\ u \sin \varphi ^{\dag }\sin \theta ^{\dag } +v \cos \varphi ^{\dag } \end{pmatrix}\\&\quad +R'(1+\epsilon z) \Omega '^2\begin{pmatrix} \sin \varphi ^{\dag }\cos \varphi ^{\dag }\cos \theta ^{\dag }\\ -\sin ^2\varphi ^{\dag } \sin \theta ^{\dag }\cos \theta ^{\dag }\\ -\cos ^2\varphi ^{\dag }\cos ^2\theta ^{\dag } -\sin ^2\theta ^{\dag } \end{pmatrix}\\&=-\frac{1}{\bar{\rho '}\rho }\begin{pmatrix} \frac{1}{R'(1+\epsilon z)\cos \theta ^{\dag }}\frac{\partial (\bar{\rho '}U'^2p)}{\partial \varphi ^{\dag }}\\ \frac{1}{R'(1+\epsilon z)}\frac{\partial (\bar{\rho '}U'^2p)}{\partial \theta ^{\dag }}\\ \frac{1}{H'} \frac{\partial (\bar{\rho '}g'H'\int _z^0\rho (s)ds+\bar{\rho '}U'^2 p)}{\partial z} \end{pmatrix} \\&\quad -\begin{pmatrix} 0\\ 0\\ g'\frac{1}{(1+\epsilon z)^2 }\\ \end{pmatrix} +\frac{U'^2}{L'}A_V \left( \frac{\partial ^2}{\partial z^2}+ \frac{2\epsilon }{(1+\epsilon z)}\frac{\partial }{\partial z}\right) \begin{pmatrix} u \\ v\\ \epsilon w\\ \end{pmatrix} \\&\quad +\frac{U'^2 H'\, A_H}{(1+\epsilon z)^2}\left( \frac{1}{ R'^2\cos ^2 \theta ^{\dag }} \frac{\partial ^2 }{\partial \varphi ^{\dag 2}}+\frac{1}{R'^2}\frac{\partial ^2}{\partial \theta ^{\dag 2}} - \frac{\tan \theta ^{\dag }}{R'^2}\frac{\partial }{\partial \theta ^{\dag }}\right) \begin{pmatrix} u \\ v\\ \epsilon w\\ \end{pmatrix}\\&\quad -\frac{U'^2 H'}{R'^2(1+\epsilon z)^2\cos ^2\theta ^{\dag }}\begin{pmatrix} A_H u\\ A_H v\\ 2\delta A_V(\epsilon w\cos ^2\theta ^{\dag }-v\sin \theta ^{\dag }\cos \theta ^{\dag }) \end{pmatrix}\\&\quad +\frac{2U'^2 H'}{R'^2(1+\epsilon z)^2}A_H\frac{\partial }{\partial \theta ^{\dag }}\begin{pmatrix} 0\\ \epsilon w\\ -v \end{pmatrix} \\&\quad +\frac{2U'^2 H'}{R'^2(1+\epsilon z)\cos \theta ^{\dag }}A_H\frac{\partial }{\partial \varphi ^{\dag }}\begin{pmatrix} \epsilon w -v \tan \theta ^{\dag }\\ u \tan \theta ^{\dag }\\ -u \end{pmatrix}. \end{aligned} \end{aligned}$$

In (14), a horizontal scaling factor is missing. To address this, we introduce a tangent plane approximation given by:

15 $$\begin{aligned} \left\{ \begin{array}{ll} x' = R' \cos \theta ^{\dag }_0 (\varphi ^{\dag } - \varphi ^{\dag }_0), \\ y' = R' (\theta ^{\dag } - \theta ^{\dag }_0), \end{array} \right. \end{aligned}$$

centered around the North Pole, where the coordinates are $\varphi _0^{\dag } = \pi /2$ and $\theta _0^{\dag } = 0$, and the scaling

16 $$\begin{aligned} x' = L'x, \qquad y' = L'y. \end{aligned}$$

Using (15) and (16), it follows that in the tangent plane approximation centered at the North Pole ($\varphi _0^{\dag } = \pi /2$, $\theta _0^{\dag } = 0$), the following transformations hold:

17 $$\begin{aligned} \frac{1}{R' \cos \theta ^{\dagger }} \frac{\partial }{\partial \varphi ^{\dagger }} \rightarrow \frac{1}{L'} \frac{\partial }{\partial x}, \qquad \frac{1}{R'} \frac{\partial }{\partial \theta ^{\dagger }} \rightarrow \frac{1}{L'} \frac{\partial }{\partial y}, \qquad \frac{\tan \theta ^{\dagger }}{R'} \frac{\partial }{\partial \theta ^{\dagger }} \rightarrow 0. \end{aligned}$$

Using (15), (16) and (17) in (14), and dividing by $U'^2/L'$ both sides leads to

18 $$\begin{aligned} \begin{aligned}&\left[ \frac{\partial }{\partial t}+ \frac{u}{(1+\epsilon z) } \frac{\partial }{\partial x} + \frac{v}{(1+\epsilon z) } \frac{\partial }{\partial y} + \frac{\epsilon }{\delta }w\frac{\partial }{\partial z} \right] \begin{pmatrix} u \\ v\\ \epsilon w\\ \end{pmatrix}\\&\quad + \frac{\epsilon }{\delta }\frac{1}{(1+\epsilon z)}\begin{pmatrix} \epsilon uw\\ \epsilon vw\\ -u^2-v^2 \end{pmatrix}+f\begin{pmatrix} -v\\ u \\ 0 \end{pmatrix}\\&=-\frac{1}{\rho } \begin{pmatrix} \frac{1}{(1+\epsilon z)}\frac{\partial p}{\partial x}\\ \frac{1}{(1+\epsilon z)}\frac{\partial p}{\partial y}\\ \frac{1}{\delta }\frac{\partial p}{\partial z} \end{pmatrix}+\begin{pmatrix} 0\\ 0\\ \frac{\epsilon }{\delta }\frac{(2z+\epsilon z^2)}{(1+\epsilon z)}\frac{1}{Fr^2} \end{pmatrix}\\&\quad +A_V \left( \frac{\partial ^2}{\partial z^2}+ \frac{2\epsilon }{(1+\epsilon z)}\frac{\partial }{\partial z}\right) \begin{pmatrix} u \\ v\\ \epsilon w\\ \end{pmatrix} + \frac{\delta }{(1+\epsilon z)^2}A_H\left( \frac{\partial ^2 }{\partial x^2}+\frac{\partial ^2}{\partial y^2} \right) \begin{pmatrix} u \\ v\\ \epsilon w\\ \end{pmatrix}\\&\quad -\frac{\epsilon ^2}{\delta (1+\epsilon z)^2}\begin{pmatrix} A_H u\\ A_H v\\ 2\epsilon \delta A_Vw \end{pmatrix}+2\frac{\epsilon }{(1+\epsilon z)^2}A_H\begin{pmatrix} \epsilon \frac{\partial w}{\partial x}\\ \epsilon \frac{\partial w}{\partial y}\\ - \frac{\partial u}{\partial x}- \frac{\partial v}{\partial y} \end{pmatrix}, \end{aligned} \end{aligned}$$

where $Fr=\frac{U'}{\sqrt{g'H'}}\approx 0.005$ is the Froude number, relating the inertia forces in a system to the effects of gravity.

Looking at the leading order terms in the three components of (18) we get the following momentum equations:

19 $$\begin{aligned} \left\{ \begin{aligned}&\frac{\partial u}{\partial t} + u \frac{\partial u}{\partial x} + v\frac{\partial u}{\partial y} - fv= -\frac{1}{\rho }\frac{\partial p}{\partial x} + A_V \frac{\partial ^2 u}{\partial z^2},\\&\frac{\partial v}{\partial t} + u \frac{\partial v}{\partial x} + v\frac{\partial v}{\partial y} +fu= -\frac{1}{\rho }\frac{\partial p}{\partial y} + A_V \frac{\partial ^2 v}{\partial z^2},\\&\frac{\partial p}{\partial z}=0. \end{aligned}\right. \end{aligned}$$

In an analogous way, applying the scaling (9) and (10), as well as the tangent plane approximation (17) to the continuity equation (8) we get

20 $$\begin{aligned} \frac{1}{1+\epsilon z}\left( \frac{\partial u}{\partial x}+\frac{\partial v}{\partial y}\right) + \frac{\epsilon }{\delta (1+\epsilon z)^2}\frac{\partial w}{\partial z} =0, \end{aligned}$$

which, at leading order, reduces to

21 $$\begin{aligned} \frac{\partial u}{\partial x} + \frac{\partial v}{\partial y}=0. \end{aligned}$$

### Boundary Conditions

Let us now describe the boundary conditions associated to the ice-drift solutions to (19) and (21).

Firstly, the velocity field ${\varvec{u}}'_{\dag }=(u'_{\dag }, v'_{\dag }, w'_{\dag })$ should posses a strong decay with depth, as ice-drift flows are negligible beneath the low-salinity surface layer (not in excess of $200\,m$). As the vertical velocity component is neglected at leading order in (19) and (21), the boundary conditions capturing the decay with depth of wind drift flows is given by

22 $$\begin{aligned} \frac{ \partial (u'^2+v'^2)}{\partial z'}<0\quad \text {for}\quad z'<0. \end{aligned}$$

Additionally, we impose on the free surface the following condition on the pressure:

23 $$\begin{aligned} p'=p'_{atm}\quad \text {on}\quad z'=0, \end{aligned}$$

where $p'_{atm}$ is the constant atmospheric pressure. The condition (23), due to (10) became

24 $$\begin{aligned} p=0\quad \text {on}\quad z=0. \end{aligned}$$

The presence of the sea-ice cover, providing a resistive force against the motion forced by the wind and therefore damping surface waves, justifies the modeling assumption of a flat free surface $z'=0$, leading to the kinematic boundary condition

25 $$\begin{aligned} w'_{\dag }=0\quad \text {on}\quad z'=0, \end{aligned}$$

representing the absence of mass flux across the free boundary.

The continuity of the stress across the interface gives the surface boundary condition

26 $$\begin{aligned} (\tau ^x,\tau ^y)=\underbrace{\rho '(0)A'_V \left( \frac{\partial u'}{\partial z'},\frac{\partial v'}{\partial z'}\right) }_{\text {shear stress}}\quad \text {on}\quad z'=0, \end{aligned}$$

relating the wind-ice-stress vector $(\tau ^x,\tau ^y)$ with the shear stress.

The wind-ice-water stress can be partitioned, in general, as (see [7])

27 $$\begin{aligned} (\tau ^x,\tau ^y)=\alpha \varvec{\tau }_{\mathrm {\textrm{ice}}}+(1-\alpha ) \varvec{\tau }_{\textrm{air}}, \end{aligned}$$

where $\alpha$ is the ice-covered surface area fraction, $\varvec{\tau }_{\textrm{ice}}$ is the ice-water stress and $\varvec{\tau }_{\textrm{air}}$ is the air-water stress. As we are considering flows in a region around the North Pole, the surface is always covered with ice ($\alpha =1$), hence equation (27) reduces to

28 $$\begin{aligned} (\tau ^x,\tau ^y)=\varvec{\tau }_{\textrm{ice}}. \end{aligned}$$

The ice-water stress $\varvec{\tau }_{\textrm{ice}}$ is given by the bulk formula (see [7] or [24])

29 $$\begin{aligned} \varvec{\tau }_{\textrm{ice}}=\rho '(0) C_{\textrm{ice}}|{\textbf{U}}'_{\textrm{ice}}-{\textbf{U}}'|({\textbf{U}}'_{\textrm{ice}}-{\textbf{U}}'), \end{aligned}$$

where $\rho '(0)\approx 1026\ kg\, m^{-3}$ is the water density at the surface, $C_{\textrm{ice}}\approx 5.5\cdot 10^{-3}$ is the ice-water drag coefficient, ${\textbf{U}}'_{\textrm{ice}}$ is the ice velocity and ${\textbf{U}}'$ is the surface current velocity.

Combining (26) and (29) gives the following boundary condition describing the horizontal momentum exchange at the surface:

30 $$\begin{aligned} \rho '(0)A_V'\left( \frac{\partial u'}{\partial z'},\frac{\partial v'}{\partial z'}\right) =\rho '(0) C_{\textrm{ice}} |{\textbf{U}}'_{\textrm{ice}}-{\textbf{U}}'|({\textbf{U}}'_{\textrm{ice}}-{\textbf{U}}') \quad \text {on}\quad z'=0, \end{aligned}$$

which becomes

31 $$\begin{aligned} \left( \frac{\partial u}{\partial z},\frac{\partial v}{\partial z}\right) =\frac{C_i}{A_V} |(u_i-u,v_i-v)|(u_i-u,v_i-v) \quad \text {on}\quad z=0, \end{aligned}$$

after non-dimensionalizing with

32 $$\begin{aligned} {\textbf{U}}'_{\textrm{ice}}=U'(u_i,v_i)\quad \text {and}\quad C_{\textrm{ice}}=\frac{H'}{L'}C_i. \end{aligned}$$

Summing up, the boundary conditions in non-dimensional form relevant to the nonlinear drift flow are

33 $$\begin{aligned} {\left\{ \begin{array}{ll} p=0& \text {on}\ z=0,\\ \frac{ \partial (u^2+v^2)}{\partial z}<0& \text {for}\ z<0,\\ \left( \frac{\partial u}{\partial z},\frac{\partial v}{\partial z}\right) =\frac{C_i}{A_V} |(u_i-u,v_i-v)|(u_i-u,v_i-v) & \text {on}\ z=0. \end{array}\right. } \end{aligned}$$

The system (19) and (21), with its simple *z*-dependence, allows for solutions that involve layered, depth-dependent 2*D* incompressible horizontal flows. This will be examined in more detail in the following section, adopting a Lagrangian formulation.

## The Nonlinear Ice-Drift Solution in Lagrangian Framework

Typically, the motion of a fluid is studied by adopting the so-called Eulerian approach, in which the motion of a fluid is described by observing it from a fixed reference frame in space. In this approach, the focus is on analyzing how the properties of the fluid (such as velocity, pressure, density, temperature, etc.) change over time at specific points in space, rather than tracking the motion of individual fluid particles [25].

There is another approach to describe the motion of a fluid, named Lagrangian, in which one follows the motion of fixed fluid particles, whereby the coordinates of such an individual particle, *x*, *y*, *z* are considered to depend on three spatial coordinates, ${\textsf{a}}, {\textsf{b}}, {\textsf{c}}$, and time *t*, called Lagrangian coordinates or material variables or labeling variables (see [26]).

**Fig. 5:**
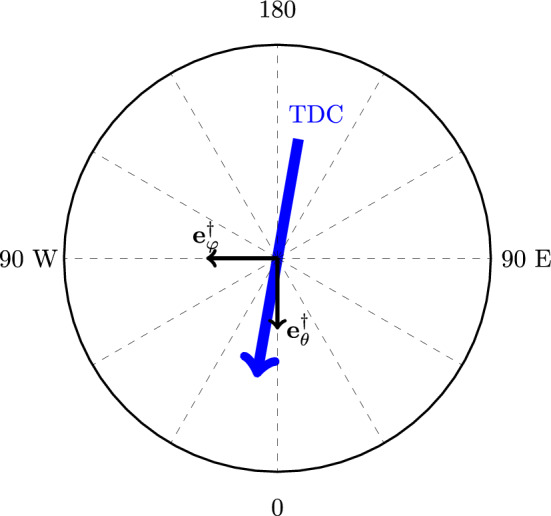
The base vector ${\textbf{e}}_{\varphi }^{\dag }, {\textbf{e}}_{\theta }^{\dag }$ of the rotated spherical coordinate system at the North Pole, and a schematic depiction of the geostrophic component of the Transpolar Drift Current (TDC)

The central part of the Arctic Ocean features the presence of the Transpolar Drift Current (TDC), which at the North Pole is steady and will be modeled as the sum of an Ekman-type spiral plus a geostrophic flow (see [5]). In our model we assume the amplitude of the Ekman-type component of the TDC to be independent of *x* and *y* and ${\mathfrak {u}}_g$ and ${\mathfrak {v}}_g$ representing the geostrophic flow (which is assumed to be constant for simplicity). Therefore, the Transpolar Drift current can be modeled as the velocity vector

34 $$\begin{aligned} \text {TDC}=\left( m e^{sz}\cos (sz+\alpha )+{\mathfrak {u}}_g,\ m e^{sz}\sin (sz+\alpha )+{\mathfrak {v}}_g\right) , \end{aligned}$$

where *m* is the amplitude of the Ekman-type spiral^2^ of the TDC at the surface, *s* is a multiplication factor representing “how fast” the Ekman-type flow should decay to zero, $\alpha$ is the angle that represents the direction of the TDC at the surface and finally ${\mathfrak {u}}_g$ and ${\mathfrak {v}}_g$ are the components of the constant geostrophic flow. See Figure 5 for an approximate depiction of the geostrophic component of the Transpolar Drift Current. As the geostrophic component of the Transpolar Drift Current should be almost parallel to the Greenwich Meridian (the meridian of $0^{\circ }$ latitude), we must require ${\mathfrak {v}}_g>{\mathfrak {u}}_g$.

Adopting the Lagrangian approach, and specifying at every time *t* the positions

35 $$\begin{aligned} \left\{ \begin{aligned} x(t; a,b,z)=a + d_1(z)t -\frac{1}{k} e ^{k(b+z)}\sin (k(a-z-ct)) \\ +{\left( m e^{sz}\cos (sz+\alpha )+{\mathfrak {u}}_g\right) t},\\ y(t; a,b,z)=b + d_2(z)t +\frac{1}{k} e ^{k(b+z)}\cos (k(a-z-ct)) \\ +\left( m e^{sz}\sin (sz+\alpha )+{\mathfrak {v}}_g\right) t, \end{aligned}\right. \end{aligned}$$

of the horizontally moving fluid particles in terms of the depth *z*, the material variables *a*, *b* and the parameters $k>0$ and $c>0$, for suitably chosen functions $d_1(z)$ and $d_2(z)$, we will show that a solution to the nonlinear ice-drift flow at the North Pole (19), (21) satisfying the boundary conditions (33) is given by (35). The time-periodic functions in (35) have principal time-period $T=\frac{2\pi }{ck}$.

The material variable $a$ is assumed to be a real number, while $b$ must satisfy $b_1< b< b_2 < 0$, ensuring that for $z \le 0$, the horizontal oscillations in (35) of a particle decay exponentially with increasing depth.

We anticipate that, due to the structure of $d(z)=(d_1(z),\, d_2(z))$, (cf. (58) and (72)), the position at every time *t* will be

36 $$\begin{aligned} \left\{ \begin{aligned} x(t; a,b,z) = a + \left[ \left( d_1(0)+ m \cos (\alpha )\right) e^{(1+i)\lambda z}+{\mathfrak {u}}_g\right] t \\ -\frac{1}{k} e ^{k(b+z)}\sin (k(a-z-ct)), \\ y(t; a,b,z) = b + \left[ \left( d_2(0)+ m \sin (\alpha )\right) e^{(1+i)\lambda z}+{\mathfrak {v}}_g\right] t \\ +\frac{1}{k} e ^{k(b+z)}\cos (k(a-z-ct)) . \end{aligned} \right. \end{aligned}$$

where $\lambda =\sqrt{\frac{f}{2 A_V}}$ is the inverse of the Ekman depth (see [25]). Namely, at each vertical level $z$ beneath the ocean surface ($z = 0$), (36) represents a depth-dependent oscillatory motion, characterized by a varying amplitude and phase shift, superimposed on a Ekman current propagating in the direction of the horizontal vector $\left( [d_1(0)+ m\cos (\alpha )]e^{(1+i)\lambda z},\ [d_2(0)+ m \sin (\alpha )]e^{(1+i)\lambda z} \right)$ and on a constant geostrophic flow $({\mathfrak {u}}_g,{\mathfrak {v}}_g)$.

For simplicity we write

37 $$\begin{aligned} \xi =k(a-z-ct), \end{aligned}$$

and it follows that, at every fixed time *t*,

38 $$\begin{aligned} \begin{pmatrix} \frac{ \partial x}{\partial a} &  \frac{ \partial x}{\partial b}\\ \frac{ \partial y}{\partial a} &  \frac{ \partial y}{\partial b} \end{pmatrix}=\begin{pmatrix} 1-e^{k(b+z)}\cos (\xi ) &  -e^{k(b+z)}\sin (\xi )\\ -e^{k(b+z)}\sin (\xi ) & 1+e^{k(b+z)}\cos (\xi ) \end{pmatrix}. \end{aligned}$$

The determinant of the matrix in (38) is equal to $1-e^{2k(b+z)}$, and its inverse is

39 $$\begin{aligned} \begin{pmatrix} \frac{ \partial a}{\partial x} &  \frac{ \partial a}{\partial y}\\ \frac{ \partial b}{\partial x} &  \frac{ \partial b }{\partial y} \end{pmatrix}=\frac{1}{1-e^{2k(b+z)}}\begin{pmatrix} 1+e^{k(b+z)}\cos (\xi ) &  e^{k(b+z)}\sin (\xi )\\ e^{k(b+z)}\sin (\xi ) & 1-e^{k(b+z)}\cos (\xi ) \end{pmatrix}. \end{aligned}$$

From (35) we have that

40 $$\begin{aligned} \left\{ \begin{aligned}&u=\frac{\partial x}{\partial t}=\overbrace{d_1(z) +c e ^{k(b+z)}\cos (\xi )+m e^{sz}\cos (sz+\alpha )}^{:={\hat{u}}}+{\mathfrak {u}}_g,\\&v=\frac{\partial y}{\partial t}=\underbrace{d_2(z) +ce ^{k(b+z)}\sin (\xi )+m e^{sz}\sin (sz+\alpha )}_{:={\hat{v}}}+{\mathfrak {v}}_g. \end{aligned}\right. \end{aligned}$$

where with ${\hat{u}}, {\hat{v}}$ we indicate the non-geostrophic components of the horizontal velocities (*u*, *v*), consisting of the Ekman flow plus oscillations. Moreover, since

41 $$\begin{aligned} \begin{aligned}&\begin{pmatrix} \frac{ \partial u}{\partial x} &  \frac{ \partial v}{\partial x}\\ \frac{ \partial u}{\partial y} &  \frac{ \partial v}{\partial y} \end{pmatrix} = \begin{pmatrix} \frac{ \partial a}{\partial x} &  \frac{ \partial b}{\partial x}\\ \frac{ \partial a}{\partial y} &  \frac{ \partial b}{\partial y} \end{pmatrix} \begin{pmatrix} \frac{ \partial u}{\partial a} &  \frac{ \partial v}{\partial a}\\ \frac{ \partial u}{\partial b} &  \frac{ \partial v}{\partial b} \end{pmatrix} \\&\ =\frac{kc}{1-e^{2k(b+z)}}\begin{pmatrix} -e^{k(b+z)}\sin (\xi )&  e^{k(b+z)}\cos (\xi ) + e^{2k(b+z)}\\ e^{k(b+z)}\cos (\xi )- e^{2k(b+z)} &  e^{k(b+z)}\sin (\xi ) \end{pmatrix}, \end{aligned} \end{aligned}$$

at each horizontal level the two-dimensional horizontal flow (35) has constant vorticity

42 $$\begin{aligned} \frac{\partial u}{\partial y}- \frac{\partial v}{\partial x}=\frac{2kc }{1-e^{-2k(b+z)}}. \end{aligned}$$

Let us decompose the pressure as

43 $$\begin{aligned} p=P_G+{\hat{p}} \end{aligned}$$

where

44 $$\begin{aligned} \left\{ \begin{aligned} \frac{\partial P_G}{\partial x}&= \rho f {\mathfrak {v}}_g,\\ \frac{\partial P_G}{\partial y}&= -\rho f {\mathfrak {u}}_g. \end{aligned}\right. \end{aligned}$$

In general, pressure gradients are balanced by the geostrophic current (see [25]), that is to say that $\frac{\partial p}{\partial x}\approx \rho f {\mathfrak {v}}_g= \frac{\partial P_G}{\partial x}$ and $\frac{\partial p}{\partial y}\approx -\rho f {\mathfrak {u}}_g=\frac{\partial P_G}{\partial y}$ where the last equalities are due to (44). For this reason we assume

45 $$\begin{aligned} \frac{\partial {\hat{p}}}{\partial x}=\frac{\partial {\hat{p}}}{\partial y}=0. \end{aligned}$$

As the first two equations in (19) are

46 $$\begin{aligned} \left\{ \begin{aligned} \frac{D u}{D t} - fv= -\frac{1}{\rho }\frac{\partial p}{\partial x} + A_V \frac{\partial ^2 u}{\partial z^2},\\ \frac{D v}{D t}+fu= -\frac{1}{\rho }\frac{\partial p}{\partial y} + A_V \frac{\partial ^2 v}{\partial z^2}, \end{aligned}\right. \end{aligned}$$

using (44) we can write

47 $$\begin{aligned} \left\{ \begin{aligned} \frac{D u}{D t} - f{\hat{v}}= -\frac{1}{\rho }\frac{\partial {\hat{p}}}{\partial x} + A_V \frac{\partial ^2 u}{\partial z^2},\\ \frac{D v}{D t}+f{\hat{u}}= -\frac{1}{\rho }\frac{\partial {\hat{p}}}{\partial y} + A_V \frac{\partial ^2 v}{\partial z^2}. \end{aligned}\right. \end{aligned}$$

which reduces to

48 $$\begin{aligned} \left\{ \begin{aligned} \frac{D u}{D t} - f{\hat{v}}= A_V \frac{\partial ^2 u}{\partial z^2},\\ \frac{D v}{D t}+f{\hat{u}}= A_V \frac{\partial ^2 v}{\partial z^2}. \end{aligned}\right. \end{aligned}$$

under the approximation (45).

Moreover, as in Lagrangian framework the material derivative corresponds to the time derivative, we have that

49 $$\begin{aligned} \left\{ \begin{aligned}&\frac{D u}{D t}=kc^2 e ^{k(b+z)}\sin (\xi ),\\&\frac{D v}{D t}=-kc^2e ^{k(b+z)}\cos (\xi ), \end{aligned}\right. \end{aligned}$$

therefore inserting (40) and (49) into (48) we get

50 $$\begin{aligned} \left\{ \begin{aligned} 0=\left[ f d_2(z)+A_V d_1''(z)+(f-2A_Vs^2)me^{sz}\sin (sz+\alpha )\right] \\ +\left( fc + 2 A_V k^2 c - k c^2\right) e^{k(b+z)}\sin (\xi ),\\ 0=-\left[ f d_1(z)-A_V d_2''(z)+ (f-2A_Vs^2)me^{sz}\cos (sz+\alpha )\right] \\ -\left( fc + 2 A_V k^2 c - k c^2\right) e^{k(b+z)}\cos (\xi ). \end{aligned}\right. \end{aligned}$$

The system (50) is satisfied if

51 $$\begin{aligned} \left\{ \begin{aligned} f d_2(z)+A_V d_1''(z)+(f-2A_Vs^2)me^{sz}\sin (sz+\alpha )=0,\\ -f d_1(z)+A_V d_2''(z)- (f-2A_Vs^2)me^{sz}\cos (sz+\alpha )=0,\\ \left( fc + 2 A_V k^2 c - k c^2\right) e^{k(b+z)}=0.\\ \end{aligned}\right. \end{aligned}$$

Therefore, given the dispersion relation

52 $$\begin{aligned} c=\frac{f}{k}+2A_V k, \end{aligned}$$

the system (50) reduces to

53 $$\begin{aligned} \left\{ \begin{aligned}&0= f d_2(z)+A_V d_1''(z)+(f-2A_Vs^2)me^{sz}\sin (sz+\alpha ),\\&0 =-f d_1(z)+A_V d_2''(z)- (f-2A_Vs^2)me^{sz}\cos (sz+\alpha ), \end{aligned}\right. \end{aligned}$$

and, writing in complex-variable notation,

54 $$\begin{aligned} d(z)=d_1(z) + i d_2(z)\qquad \text {and}\qquad {\mathcal {K}}=m\frac{f-2A_Vs^2}{A_V}=2m(\lambda ^2-s^2), \end{aligned}$$

the system (53) becomes

55 $$\begin{aligned} d''(z)-i\frac{f}{A_V} d(z)=i{\mathcal {K}}e^{i\alpha }e^{(1+i)sz}. \end{aligned}$$

If $s=\lambda$, ${\mathcal {K}}=0$, the solution is given by

56 $$\begin{aligned} d(z)=A_1 e^{(1+i)\lambda z}+ B_1e^{-(1+i)\lambda z}=A_1 e^{(1+i)\lambda z}+ B_1e^{-(1+i)\lambda z}, \end{aligned}$$

where $\lambda =\sqrt{\frac{f}{2A_V}}$ and $A_1, B_1 \in {\mathbb {C}}$ are complex constants. On the other hand, if $s\not =\lambda$, given two complex constants $A_2, B_2 \in {\mathbb {C}}$, the solution is

57 $$\begin{aligned} \begin{aligned} d(z)&=A_2 e^{(1+i)\lambda z}+ B_2e^{-(1+i)\lambda z}+ \frac{{\mathcal {K}}}{2(s^2-\lambda ^2)}e^{i\alpha }e^{(1+i)sz}=\\&=A_2 e^{(1+i)\lambda z}+ B_2e^{-(1+i)\lambda z}-me^{i\alpha }e^{(1+i)sz}. \end{aligned} \end{aligned}$$

As wind drift currents are insignificant at great depth, we need to set $B_1=B_2=0$, and equaling the previous two equations for *d*(*z*) at $z=0$ gives $A_1=A_2-me^{i\alpha }$, finally leading to

58 $$\begin{aligned} d(z)=(d(0)+me^{i\alpha }) e^{(1+i)\lambda z}-me^{i\alpha }e^{(1+i)sz},\qquad \forall \,s\in {\mathbb {R}}^+ , \end{aligned}$$

with $d(0)=d_1(0)+id_2(0)\in {\mathbb {C}}$ being the the mean ice-drift current at $z=0$, and $(m\cos (\alpha )+{\mathfrak {u}}_g, m\sin (\alpha )+{\mathfrak {v}}_g)=:({\mathfrak {u}}_{TDC}, {\mathfrak {v}}_{TDC})=:{\mathfrak {U}}_{TDC}$ representing the Transpolar Drift Current at the surface.

The time-average of the velocity (40), which in view of (58) reads as

59 $$\begin{aligned} \begin{aligned} u+iv&=\overbrace{(d_1(0)+m\cos (\alpha ))e^{(1+i)\lambda z}+i(d_2(0)+m\sin (\alpha ))e^{(1+i)\lambda z}}^{d(z)=d_1(z)+id_2(z)}+\\&\ +c e^{k(b+z)}e^{ik(a-z-ct)}+({\mathfrak {u}}_g+i{\mathfrak {v}}_g), \end{aligned} \end{aligned}$$

over a period $T=\frac{2\pi }{ck}=\frac{2\pi }{f+2A_Vk^2}$ yields the mean-drift current, which is, due to (58), an Ekman spiral *d*(*z*) plus the geostrophic component ${\mathfrak {U}}_{g}={\mathfrak {u}}_g+i{\mathfrak {v}}_g$ of the TDC

60 $$\begin{aligned} \langle u+iv\rangle _T=(d_1(0)+id_2(0) +m\cos (\alpha )+i\,m\sin (\alpha ))e^{(1+i)\lambda z}+{\mathfrak {u}}_g+i{\mathfrak {v}}_g=d(z)+{\mathfrak {U}}_{g}, \end{aligned}$$

where $\langle \cdot \rangle _T$ represents the time-average.

Due to the structure of the mean current (60), the boundary condition (31) reduces to

61 $$\begin{aligned} \frac{\lambda A_V}{C_i} \left( {\mathcal {D}}_1-{\mathcal {D}}_2, {\mathcal {D}}_1+{\mathcal {D}}_2\right) = |(u_i-{\mathfrak {u}}_g-{\mathcal {D}}_1,v_i-{\mathfrak {v}}_g-{\mathcal {D}}_2)|(u_i-{\mathfrak {u}}_g-{\mathcal {D}}_1 ,v_i-{\mathfrak {v}}_g-{\mathcal {D}}_2), \end{aligned}$$

in which the unknown vector components ${\mathcal {D}}_1=d_1(0)+m\cos (\alpha )$ and ${\mathcal {D}}_2=d_2(0)+m\sin (\alpha )$ represent the non-dimensional ice-drift non-geostrophic surface current. Writing

62 $$\begin{aligned} R e^{i \theta }=(u_i-{\mathfrak {u}}_g-{\mathcal {D}}_1)+i(v_i-{\mathfrak {v}}_g-{\mathcal {D}}_2), \end{aligned}$$

(61) can be rewritten as

63 $$\begin{aligned} \beta \left[ ({\mathcal {D}}_1-{\mathcal {D}}_2)+i( {\mathcal {D}}_1+{\mathcal {D}}_2)\right] =R^2 e^{i \theta }, \end{aligned}$$

or equivalently, as

64 $$\begin{aligned} R^2 e^{i \theta }+\beta (1+i) R e^{i \theta }=\zeta , \end{aligned}$$

with $\beta =\frac{\lambda A_V}{C_i}>0$, for the unknowns $R\ge 0$ and $\theta \in [0,2\pi )$, where

65 $$\begin{aligned} \zeta =\beta [(u_i-{\mathfrak {u}}_g-v_i+{\mathfrak {v}}_g)+i(u_i-{\mathfrak {u}}_g+v_i-{\mathfrak {u}}_g)] \in {\mathbb {C}}. \end{aligned}$$

As the ice moves faster than the Trasnpolar Drift Current (see [5]), $\zeta \not =0$. We therefore infer from (64) that $R\not =0$ and

66 $$\begin{aligned} e^{i \theta }=\frac{ \zeta }{R[R+\beta (1+i)]}. \end{aligned}$$

Multiplying (66) by its complex conjugate gives the quartic equation

67 $$\begin{aligned} {\mathcal {P}}(R)=R^4+2\beta R^3+2\beta ^2R^2-|\zeta |^2=0, \end{aligned}$$

and, since ${\mathcal {P}}(0)<0$, $\lim _{R\rightarrow \pm \infty } {\mathcal {P}}(R)=+\infty$ and ${\mathcal {P}}''(R)>0$, ${\mathcal {P}}$ is strictly convex, ensuring that there is one solution $R>0$ of (67).

We remark that our analysis shows that the boundary condition (31) uniquely determines the surface current *d*(0) knowing the sea-ice velocity and the TDC velocity ${\mathfrak {U}}_{TDC}$ at the surface.

Writing $(u_i-{\mathfrak {u}}_g)+i (v_i-{\mathfrak {v}}_g)={\mathcal {U}}e^{i\phi }$ with $\phi \in [0,2\pi )$ and ${\mathcal {U}}>0$, and ${\mathcal {D}}_1+i{\mathcal {D}}_2 ={\mathcal {D}}e^{i\psi }$ with $\psi \in [0,2\pi )$ and ${\mathcal {D}}>0$, $R=|{\mathcal {U}}e^{i\phi }-{\mathcal {D}}e^{i\psi }|>0$ and (61) can be rewritten as

68 $$\begin{aligned} {\mathcal {U}}e^{i(\phi -\psi )}={\mathcal {D}}+\beta \frac{(1+i){\mathcal {D}}}{|{\mathcal {U}}e^{i\phi }-{\mathcal {D}}e^{i\psi }|}. \end{aligned}$$

As the right-hand side of (68) is a complex number with real part bigger than the (positive) imaginary part, it follows that $0<\phi -\psi <\nicefrac {\pi }{4}$.

Writing the ice velocity ${\textbf{U}}_{\textrm{ice}}=u_i+i\,v_i$ as

69 $$\begin{aligned} {\textbf{U}}_{\textrm{ice}}={\mathfrak {U}}_{TDC}+{\mathcal {V}}_{\textrm{rel}}={\mathfrak {U}}_{g}+(m\cos \alpha +i m\sin \alpha )+{\mathcal {V}}_{\textrm{rel}}, \end{aligned}$$

where ${\mathcal {V}}_{\textrm{rel}}$ is the vector of the ice velocity relative to the TDC, we have that

70 $$\begin{aligned} {\mathcal {U}}e^{i\phi }= {\textbf{U}}_{\textrm{ice}}-{\mathfrak {U}}_{g}=(m\cos \alpha +i m\sin \alpha )+{\mathcal {V}}_{\textrm{rel}}. \end{aligned}$$

As we have defined ${\mathcal {D}}e^{i\psi }$ as

71 $$\begin{aligned} {\mathcal {D}}e^{i\psi }= \left( d_1(0)+id_2(0)\right) +\left( m\cos (\alpha )+im\sin (\alpha )\right) , \end{aligned}$$

the fact that $0<\phi -\psi <\nicefrac {\pi }{4}$ shows that the surface current vector $\left( d_1(0)+id_2(0)\right)$ is directed to the right of the ice moving relatively to the TDC, forming an angle of less than $45^{\circ }$ with its path.

Finally, observe that, for any given $k>0$, the horizontal particle path given by (35) coupled with (58) is

72 $$\begin{aligned} \begin{aligned} x(t;a,b,z)+iy(t;a,b,z)=(a+ib)+\bigg [(d(0)+me^{i\alpha })e^{(1+i)\lambda z}\\ +{\mathfrak {U}}_{g}\bigg ]t+ \frac{1}{k}e^{k(b+z)}e^{i\left( \frac{\pi }{2}+k(a-z)-(f+2A_Vk^2)t\right) }, \end{aligned} \end{aligned}$$

representing trochoids, and is a solution of (19) and (21).

The mean-drift current, over a period $T=\frac{2\pi }{ck}=\frac{2\pi }{f+2A_Vk^2}$,

73 $$\begin{aligned} \begin{aligned} \langle u+iv\rangle _T&=\frac{1}{T}\int _0^T\left( u(t;a,b,z)+iv(t;a,b,z)\right) dt\\&=(d(0)+me^{i\alpha })e^{(1+i)\lambda z}+{\mathfrak {U}}_{g} \end{aligned} \end{aligned}$$

is the superposition of a classical Ekman spiral and a geostrophic current, while the oscillatory perturbation of the current

74 $$\begin{aligned} \frac{1}{k}e^{k(b+z)}e^{i\left( \frac{\pi }{2}+k(a-z)-(f+2A_Vk^2)t\right) } \end{aligned}$$

is essentially inertial, as $A_V={\mathcal {O}}(1)$ and $k<<1$ gives

75 $$\begin{aligned} f+2A_Vk^2\approx f \end{aligned}$$

for the frequency. Averaging over the depth the Ekman component of (73) gives the depth-averaged mean-drift Ekman of the solution (72), also known as (time-averaged) Ekman trasport,

76 $$\begin{aligned} {\mathcal {I}}_{Ek}:=\int _{-\infty }^0 (d(0)+me^{i\alpha })e^{(1+i)\lambda z}\, dz=\frac{(d(0)+me^{i\alpha })}{\lambda \sqrt{2}}e^{-i\frac{\pi }{4}}, \end{aligned}$$

which is at $45^{\circ }$ to the right of the Ekman surface current $d(0)+me^{i\alpha }$. Given that $d(0)+me^{i\alpha }$ is directed to the right of ${\textbf{U}}_{\textrm{ice}}-{\mathfrak {U}}_g$ (due to (68)), as a consequence we have that ${\mathcal {I}}_{Ek}$ is directed to the right of ${\textbf{U}}_{\textrm{ice}}-{\mathfrak {U}}_g$ with an angle between $45^{\circ }$ and $90^{\circ }$.

Observe that, for every fixed value of *z*, the particle path (72) represents a trochoid, the only known solution for the 2*D* incompressible Euler equations with free boundary discovered by Gerstner (see [27] and [26]). Therefore, the solution (72) of the governing equation for arctic ice-drift flow represents a superposition of an Ekman spiral, a Gerstner-type solution and a geostrophic current.

## Discussion

Our analysis provided an explicit solution to the nonlinear governing equations describing the leading-order dynamics of ice-drift flows in a region centered around the North Pole. Such governing equations have been obtained from the Navier-Stokes and continuity equations adopting a thin-shell approximation coupled with a tangent plane approximation. The solution, in the Lagrangian formalism, highlights the superposition of near-inertial oscillations on a mean flow, the latter being the sum of an Ekman spiral (partially driven by the Transpolar Drift Current’s Ekman-type spiral and partially driven by ice stress) and of the geostrophic component of the TDC. This solution extends the one found in [7] and [8] for regions outside the Amundsen Basin, where the Transpolar Drift Current is not present:

77 $$\begin{aligned} {\tilde{u}}+i{\tilde{v}}=(\tilde{d}_1(0)+i{\tilde{d}}_2(0))e^{(1+i)\lambda z}+c e^{k(b+z)}e^{ik(a-z-ct)}. \end{aligned}$$

In (77), the entirety of the “forcing” velocity (namely the velocity of ice or that of the wind, or a combination of them, as regions partially covered by ice where considered in [7] and [8]) induced the Ekman spiral $({\tilde{d}}_1(0)+i{\tilde{d}}_2(0))e^{(1+i)\lambda z}$. Instead in our analysis, it is shown that only a fraction of the ice-velocity (namely the velocity of ice with respect of the TDC) acts as a forcing for the ice-drift component of the Ekman spiral $({d}_1(0)+i{d}_2(0))e^{(1+i)\lambda z}$ in

78 $$\begin{aligned} \begin{aligned} u+iv=(d_1(0)+m\cos (\alpha ))e^{(1+i)\lambda z}+i(d_2(0)+m\sin (\alpha ))e^{(1+i)\lambda z}\\ +c e^{k(b+z)}e^{ik(a-z-ct)}+({\mathfrak {u}}_g+i{\mathfrak {v}}_g), \end{aligned} \end{aligned}$$

thus implying that the amplitude of the nonlinear ice-drift current at the North Pole is smaller that in other arctic regions (for the same surface stress), due to the presence of the Transpolar Drift Current.

Moreover, note that the coordinates used in [7] and [8] are the standard Cartesian coordinates for the tangent plane approximation, based on the classical spherical coordinates (*x* pointing to East and *y* pointing to North), while our analysis is built upon the use of the “new” rotated spherical coordinates developed in [4], and here reviewed in Appendix A.2.2. In spite of this difference, the qualitative properties of the solution we found in our analysis and the ones in [7] match.

Finally, our solution (36) shows an interesting feature: in (34) we described the Ekman-type part of the Transpolar Drift Current as

79 $$\begin{aligned} me^{i\alpha }e^{(1+i)sz} \end{aligned}$$

with $s\in {\mathbb {R}}^+$, but in the nonlinear solution (36) and subsequently in (58) instead of *s*, the exponent features the presence of $\lambda$ (namely the inverse of the Ekman depth), as a result of the pressure gradient being balanced by the geostrophic current in (19), leading to the system of ODEs (50). Consequently, if $s\not =\lambda$ we can conclude that only the value of the Ekman-type current of the TDC at the surface influenced the Ekman spiral of the nonlinear ice-drift flow. On the other hand, if the Ekman-type current of the TDC is a “pure” Ekman spiral, i.e. $s=\lambda$, the entirety of it is present in the solution. This result is a consequence of the fact that $\left( m e^{\lambda z}\cos (\lambda z+\alpha ),\ m e^{\lambda z}\sin (\lambda z+\alpha )\right)$ is a solution of the “classical” system of differential equations for the Ekman spiral (see e.g. [25] or [28])

80 $$\begin{aligned} \left\{ \begin{aligned} - f{\textsf{v}}= A_V \frac{\partial ^2 {\textsf{u}}}{\partial z^2},\\ f{\textsf{u}}= A_V \frac{\partial ^2 {\textsf{v}}}{\partial z^2}, \end{aligned}\right. \end{aligned}$$

recalling that $\lambda =\sqrt{\frac{f}{2A_V}}$. This, applied to (48), leads (50) to became

81 $$\begin{aligned} \left\{ \begin{aligned} 0&=f d_2(z)+A_V d_1''(z)+\left( fc + 2 A_V k^2 c - k c^2\right) e^{k(b+z)}\sin (\xi ),\\ 0&=-f d_1(z)+A_V d_2''(z) -\left( fc + 2 A_V k^2 c - k c^2\right) e^{k(b+z)}\cos (\xi ), \end{aligned}\right. \end{aligned}$$

whose solution is $d(z)=(d_1(0)e^{(1+i)\lambda z}, d_2(0)e^{(1+i)\lambda z})$, provided the dispersion relation (52). Inserting such a solution into (35) gives exactly the same expression for the solution to (19) and (21), namely

82 $$\begin{aligned} \left\{ \begin{aligned} x(t; a,b,z)&= a + \left[ \left( d_1(0)+ m \cos (\alpha )\right) e^{(1+i)\lambda z}+{\mathfrak {u}}_g\right] t -\\&\hspace{3cm}-\frac{1}{k} e ^{k(b+z)}\sin (k(a-z-ct)), \\ y(t; a,b,z)&= b + \left[ \left( d_2(0)+ m \sin (\alpha )\right) e^{(1+i)\lambda z}+{\mathfrak {v}}_g\right] t +\\&\hspace{3cm} +\frac{1}{k} e ^{k(b+z)}\cos (k(a-z-ct)) . \end{aligned} \right. \end{aligned}$$

Therefore we can state that only if $s=\lambda$, the whole Ekman component of the TDC is present in the solution of the nonlinear ice-drift problem.

The structure of (50) also shows that, if a non-geostrophic constant current is present, it is forced to “transform” into an Ekman spiral, provided that the pressure perturbation ${\hat{p}}$ or its gradient to be zero. More precisely, assuming

83 $$\begin{aligned} \left\{ \begin{aligned} x(t; a,b,z)=a + d_1(z)t -\frac{1}{k} e ^{k(b+z)}\sin (k(a-z-ct)) + \overline{{\mathfrak {u}}}t\\ y(t; a,b,z)=b + d_2(z)t +\frac{1}{k} e ^{k(b+z)}\cos (k(a-z-ct)) +\overline{{\mathfrak {v}}}t \end{aligned}\right. \end{aligned}$$

with $\overline{{\mathfrak {u}}}, \overline{{\mathfrak {v}}}$ representing a non-geostrophic constant current. We have omitted the TDC for simplicity. Given the dispersion relation (52) and repeating the same arguments as in the previous section and assuming again the pressure perturbation ${\hat{p}}$ or its gradient to be zero, we get the system

84 $$\begin{aligned} \left\{ \begin{aligned}&A_V d_1''(z)+f d_2(z)=-f\overline{{\mathfrak {v}}},\\&A_V d_2''(z)-f d_1(z)=f\overline{{\mathfrak {u}}}, \end{aligned}\right. \end{aligned}$$

which, in complex-variable notation,

85 $$\begin{aligned} d(z)=d_1(z) + i d_2(z)\qquad \text {and}\qquad \gamma :=-\frac{f}{A_V}\overline{{\mathfrak {v}}} + i\frac{f}{A_V}\overline{{\mathfrak {u}}} \end{aligned}$$

is equivalent to

86 $$\begin{aligned} d''(z)-i\frac{f}{A_V} d(z)=\gamma . \end{aligned}$$

The solution to (86), recalling that ice-drift currents are insignificant at great depth, is

87 $$\begin{aligned} d(z)=[d(0)+\overline{{\mathfrak {u}}}+i\overline{{\mathfrak {v}}}]e^{(1+i)\lambda z}-\overline{{\mathfrak {u}}}-i\overline{{\mathfrak {v}}}, \end{aligned}$$

where $d(0)\in {\mathbb {C}}$ is the ice-drift current at the surface. Therefore (83) becomes

88 $$\begin{aligned} \left\{ \begin{aligned} x(t; a,b,z)=a + [d_1(0)+\overline{{\mathfrak {u}}}]e^{(1+i)\lambda z}t -\frac{1}{k} e ^{k(b+z)}\sin (k(a-z-ct)), \\ y(t; a,b,z)=b + [d_2(0)+\overline{{\mathfrak {v}}}]e^{(1+i)\lambda z}t +\frac{1}{k} e ^{k(b+z)}\cos (k(a-z-ct)) , \end{aligned}\right. \end{aligned}$$

showing that the non-geostrophic current $\overline{{\mathfrak {u}}}, \overline{{\mathfrak {v}}}$ is forced to “transform” into an Ekman spiral.

## Figures and Tables

**Fig. 6:**
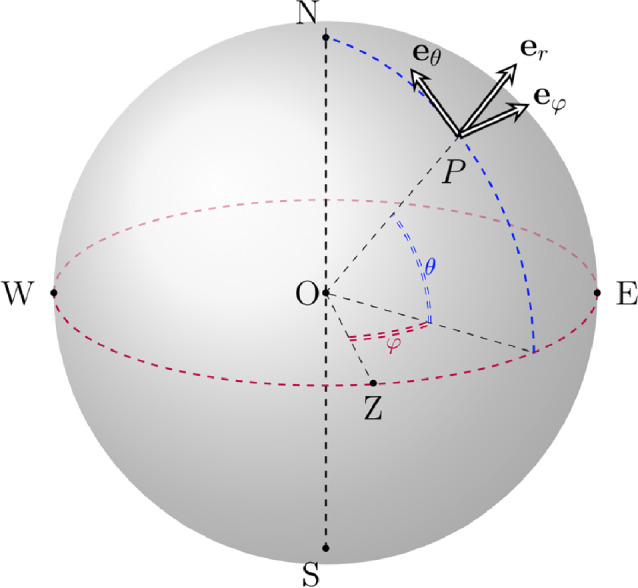
Classical spherical coordinate system. Image from [4]. CC BY 4.0 (http://creativecommons.org/licenses/by/4.0)

**Fig. 7:**
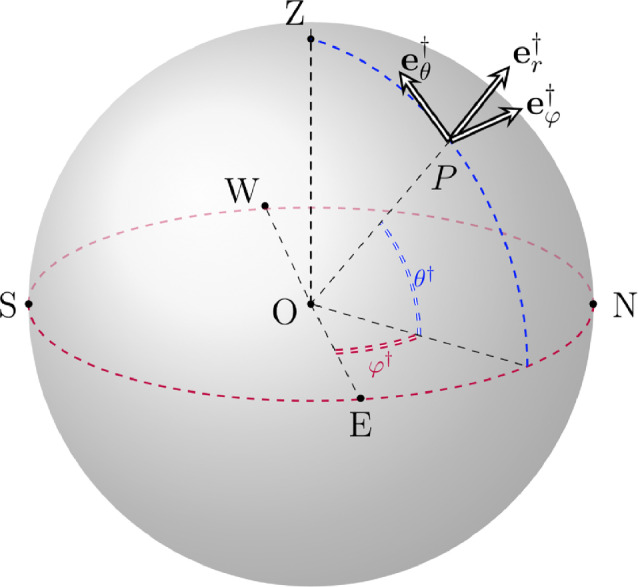
Rotated spherical coordinate system. Image from [4]. CC BY 4.0 (http://creativecommons.org/licenses/by/4.0)

## Data Availability

Not applicable.

## Appendix A Full Derivation of the Navier-Stokes Equations

This appendix is devoted to the derivation of the Navier-Stokes equation in spherical coordinates for geophysical flows (atmospheric or oceanic) in its most general setting, correcting the ones in the literature, e.g. in [4] or [9], in order to provide the most complete formulation of the governing equations.

The only assumption will be that the vertical and horizontal eddy viscosities $\mu _V$ and $\mu _H$ vary with depth but not with respect to the horizontal coordinates, which is physically reasonable since, in most cases, the scales on which the fluid flow is analyzed are much smaller than those on which eddy viscosities vary horizontally. However, extending this to account for viscosities that vary with horizontal coordinates will be almost straightforward, at the price of making the equations more intricate.

Before proceeding with the derivation, we briefly recall the formulae for the differential operators in orthogonal curvilinear coordinates used in this paper, namely the gradient and the Laplacian of a scalar field, the divergence of a vector field and of a second order tensor, the gradient of a vector field and the advection operator ${\varvec{u}}\cdot \nabla$. We refer to [29, 30], and [31] for a more complete exposition.

### A.1 Differential Operators in Orthogonal Curvilinear Coordinates

In this subsection, we will not make the distinction between physical variables (previously denoted with $'$) and non-dimensional variables. For simplicity, everything will be denoted without primes.

Let us start by setting $({\textbf{e}}_{1}, {\textbf{e}}_{2}, {\textbf{e}}_{3})$ a basis of ${\mathbb {R}}^3$, and Cartesian coordinates (*x*, *y*, *z*). A curvilinear coordinate system $u_1, u_2, u_3$ of ${\mathbb {R}}^3$ is given by the scalar functions

A1 $$\begin{aligned} \left\{ \begin{array}{ll} u_1=u_1(x,y,z),\\ u_2=u_2(x,y,z),\\ u_3=u_3(x,y,z), \end{array}\right. \qquad \left\{ \begin{array}{ll} x=x(u_1, u_2, u_3),\\ y=y(u_1, u_2, u_3),\\ z=z(u_1, u_2, u_3),\\ \end{array}\right. \end{aligned}$$

giving the so-called change of coordinates and the inverse change of coordinates. The functions in (A1) are assumed to be single-valued and to have continuous derivatives so that there is a one-to-one correspondence between (*x*, *y*, *z*) and $(u_1, u_2, u_3)$.

Let ${\mathfrak {r}}=x\,{\textbf{e}}_{1}+y\,{\textbf{e}}_{2}+z\,{\textbf{e}}_{3}$ be the position vector of a given point *P*, which, due to (A1) can be written as ${\mathfrak {r}}=x(u_1, u_2, u_3){\textbf{e}}_{1}+y(u_1, u_2, u_3){\textbf{e}}_{2}+z(u_1, u_2, u_3){\textbf{e}}_{3}$, namely ${\mathfrak {r}}={\mathfrak {r}}(u_1, u_2, u_3)$, and let

A2 $$\begin{aligned} {\textsf{h}}_i=\left| \frac{\partial {\mathfrak {r}}}{\partial u_i} \right| , \end{aligned}$$

denote the scale factors (also known as metric coefficients or Lamé coefficients). The associated unit tangent vectors are given by ${\mathfrak {e}}_i=\nicefrac {\frac{\partial {\mathfrak {r}}}{\partial u_i}}{|\frac{\partial {\mathfrak {r}}}{\partial u_i}|}=\nicefrac {\frac{\partial {\mathfrak {r}}}{\partial u_i}}{{\textsf{h}}_i}$, for $i=1,2,3$.

The aforementioned formulae for the differential operators are summarized below.

- Gradient and Laplacian of a scalar field: given a scalar field $f(u_1,u_2,u_3)$, its gradient is given by
  A3 $$\begin{aligned} \nabla f=\sum _{i}\frac{1}{{\textsf{h}}_i} \frac{\partial f}{\partial u_i}{{\mathfrak {e}}_i} \end{aligned}$$
  and its Laplacian by
  A4 $$\begin{aligned} \Delta f= \frac{1}{{\textsf{h}}}\left[ \frac{\partial }{\partial u_1}\left( \frac{{\textsf{h}}_2{\textsf{h}}_3 }{ {\textsf{h}}_1}\frac{\partial f}{\partial u_1}\right) +\frac{\partial }{\partial u_2}\left( \frac{{\textsf{h}}_1{\textsf{h}}_3 }{ {\textsf{h}}_2}\frac{\partial f}{\partial u_2}\right) + \frac{\partial }{\partial u_3}\left( \frac{{\textsf{h}}_1{\textsf{h}}_2 }{ {\textsf{h}}_3}\frac{\partial f}{\partial u_3}\right) \right] . \end{aligned}$$
- Divergence and gradient of a vector field: given a vector field $V(u_1,u_2,u_3)=\left( V_1(u_1,u_2,u_3),\right. \left. V_2(u_1,u_2,u_3), V_3 (u_1,u_2,u_3)\right)$ with respect to the base ${\mathfrak {e}}_i$ the divergence of *V* is a scalar given by
  A5 $$\begin{aligned} \begin{aligned} \nabla \cdot V=\sum _{i=1,2,3} \frac{{\textsf{h}}_i^2}{{\textsf{h}}}\nabla u_i\cdot \nabla \left( \frac{{\textsf{h}}}{{\textsf{h}}_i} V_i \right) =\frac{1}{{\textsf{h}}}\sum _{i=1,2,3} \frac{\partial }{\partial u_i}\left( \frac{{\textsf{h}}}{{\textsf{h}}_i} V_i \right) , \end{aligned} \end{aligned}$$
  while its gradient, which is a second-order tensor (see [30]), is given by the relation $(\nabla V)^T=\nabla \otimes V$ where
  A6 $$\begin{aligned} \nabla \otimes V=\sum _{i=1,2,3} \frac{{\mathfrak {e}}_i}{{\textsf{h}}_i}\frac{\partial \left( \sum _{j} V_j{\mathfrak {e}}_j\right) }{\partial u_i}. \end{aligned}$$
- Advection operator ${\varvec{u}}\cdot \nabla$: in previous sections, we wrote the velocity vector field ${\varvec{u}}$ as ${\varvec{u}}=(u,v,w)$. However, this notation in the following computations could be confusing, therefore let us write ${\varvec{u}}=({\mathfrak {U}}_1, {\mathfrak {U}}_2, {\mathfrak {U}}_3)$. Given a vector field *V*, the components of the advection operator ${\varvec{u}}\cdot \nabla$ acting on a vector field *V* are given by
  A7 $$\begin{aligned} \begin{aligned} \left\{ ({\varvec{u}}\cdot \nabla ) V\right\} _i=\sum _{j=1,2,3}\left( \frac{\mathfrak {U_j}}{{\textsf{h}}_j}\frac{\partial V_i}{\partial u_j}-\frac{\mathfrak {U_j} V_j}{{\textsf{h}}_i{\textsf{h}}_j}\frac{\partial {\textsf{h}}_j}{\partial u_i}+\frac{\mathfrak {U_i} V_j}{{\textsf{h}}_i{\textsf{h}}_j}\frac{\partial {\textsf{h}}_i}{\partial u_j}\right) . \end{aligned} \end{aligned}$$
- Divergence of a second-order tensor: the formula for the divergence of a second-order tensor ${\textbf{A}}=A^{ij}{\mathfrak {e}}_i \otimes {\mathfrak {e}}_j$ is given by (see [30])
  A8 $$\begin{aligned} \nabla \cdot {\textbf{A}}=\sum _{i,j}\left( \frac{1}{{\textsf{h}}_j}\frac{\partial A^{ij}}{\partial u_j}{\mathfrak {e}}_j + A^{ij}\left( \frac{\omega _j}{{\textsf{h}}_j}\times {\mathfrak {e}}_i\right) +\sum _k A^{ij}\left[ \frac{\omega _k}{{\textsf{h}}_k}\cdot ({\mathfrak {e}}_i\times {\mathfrak {e}}_k)\right] {\mathfrak {e}}_i\right) , \end{aligned}$$
  which, written component by component, reads as
  A9 $$\begin{aligned} \begin{aligned} (\nabla \cdot {\textbf{A}})_1&= \frac{1}{{\textsf{h}}_1} \frac{\partial A^{11}}{\partial u_1}+\frac{1}{{\textsf{h}}_2} \frac{\partial A^{12}}{\partial u_2}+\frac{1}{{\textsf{h}}_3} \frac{\partial A^{13}}{\partial u_3} \\&\quad +\frac{A^{13} + A^{31}}{{\textsf{h}}_1 {\textsf{h}}_3}\frac{\partial {\textsf{h}}_1}{\partial u_3}+\frac{A^{12} + A^{21}}{{\textsf{h}}_1{\textsf{h}}_2} \frac{\partial {\textsf{h}}_1}{\partial u_2}+\frac{A^{13}}{{\textsf{h}}_2{\textsf{h}}_3} \frac{\partial {\textsf{h}}_2}{\partial u_3}\\&\quad + \frac{A^{11} - A^{22}}{{\textsf{h}}_1{\textsf{h}}_2}\frac{\partial {\textsf{h}}_2}{\partial u_1} + \frac{A^{12} }{{\textsf{h}}_3{\textsf{h}}_2}\frac{\partial {\textsf{h}}_3}{\partial u_2}- \frac{A^{33} - A^{11}}{{\textsf{h}}_3{\textsf{h}}_1}\frac{\partial {\textsf{h}}_3}{\partial u_1} \\ (\nabla \cdot {\textbf{A}})_2&= \frac{1}{{\textsf{h}}_1} \frac{\partial A^{21}}{\partial u_1}+\frac{1}{{\textsf{h}}_2} \frac{\partial A^{22}}{\partial u_2}+\frac{1}{{\textsf{h}}_3} \frac{\partial A^{23}}{\partial u_3} \\&\quad + \frac{A^{23}}{{\textsf{h}}_1 {\textsf{h}}_3}\frac{\partial {\textsf{h}}_1}{\partial u_3}+\frac{A^{22} - A^{11}}{{\textsf{h}}_1{\textsf{h}}_2} \frac{\partial {\textsf{h}}_1}{\partial u_2}+\frac{A^{23}+A^{32}}{{\textsf{h}}_2{\textsf{h}}_3} \frac{\partial {\textsf{h}}_2}{\partial u_3}+ \\&\quad +\frac{A^{12} +A^{21}}{{\textsf{h}}_1{\textsf{h}}_2}\frac{\partial {\textsf{h}}_2}{\partial u_1} + \frac{A^{22} -A^{33}}{{\textsf{h}}_3{\textsf{h}}_2}\frac{\partial {\textsf{h}}_3}{\partial u_2}+\frac{A^{21}}{{\textsf{h}}_3{\textsf{h}}_1}\frac{\partial {\textsf{h}}_3}{\partial u_1} \\ (\nabla \cdot {\textbf{A}})_3&= \frac{1}{{\textsf{h}}_1} \frac{\partial A^{31}}{\partial u_1} +\frac{1}{{\textsf{h}}_2} \frac{\partial A^{32}}{\partial u_2}+\frac{1}{{\textsf{h}}_3} \frac{\partial A^{33}}{\partial u_3} \\&\quad + \frac{A^{33} - A^{11}}{{\textsf{h}}_1 {\textsf{h}}_3}\frac{\partial {\textsf{h}}_1}{\partial u_3}+\frac{A^{32}}{{\textsf{h}}_1{\textsf{h}}_2} \frac{\partial {\textsf{h}}_1}{\partial u_2}+\frac{A^{33}-A^{22}}{{\textsf{h}}_2{\textsf{h}}_3} \frac{\partial {\textsf{h}}_2}{\partial u_3} \\&\quad + \frac{A^{31}}{{\textsf{h}}_1{\textsf{h}}_2}\frac{\partial {\textsf{h}}_2}{\partial u_1} + \frac{A^{23}+A^{32} }{{\textsf{h}}_3{\textsf{h}}_2}\frac{\partial {\textsf{h}}_3}{\partial u_2}+ \frac{A^{13} + A^{31}}{{\textsf{h}}_3{\textsf{h}}_1}\frac{\partial {\textsf{h}}_3}{\partial u_1}. \end{aligned} \end{aligned}$$

### A.2 Equations of Motion

Now we turn to the derivation of the equations of motion.

Newton’s second law, relating the acceleration to the net force at a point, sometimes also called Cauchy’s equation of motion in the context of continuum mechanics, in vectorial (coordinate-free) form is given by [25]:

A10 $$\begin{aligned} \rho ' \frac{D {\textbf{u}}'}{D t'} = \rho ' {\textbf{g}}' + \nabla ' \cdot \varvec{\sigma }' , \end{aligned}$$

while the continuity equation is

A11 $$\begin{aligned} \frac{1}{\rho '} \frac{D \rho '}{D t'} + \nabla ' \cdot \mathbf {u'} = 0 , \end{aligned}$$

where $t'$ is the time, $\rho '$ is the fluid density, $\frac{D}{Dt'}=\frac{\partial }{\partial t' }+{\textbf{u}}'\cdot \nabla '$ is the advective derivative, ${\textbf{g}}'$ is the gravity acceleration (its modulus being approximately $9.81\, m\, s^{-2}$ at the surface of the Earth) and $\varvec{\sigma }'$ is the stress tensor (a second-order tensor with nine components that describes the internal forces within a solid body under external loads. It comprises three normal stresses and six shear stresses). Recall that we use primes to represent dimensional (physical) variables.

In the standard Cartesian coordinates system (*x*, *y*, *z*) (with respect to the basis $(\mathbf {e_1}, \mathbf {e_2}, \mathbf {e_3})$ of ${\mathbb {R}}^3$), the stress tensor’s components, for $i,j=1,2,3$, are given by the constitutive equations:

A12 $$\begin{aligned} \varvec{\sigma }'_{ij} = - \left( p' + \frac{2}{3} \mu ' \nabla ' \cdot {\textbf{u}}'\right) \delta _{ij} + 2 \mu ' \varvec{\epsilon }'_{ij}, \end{aligned}$$

with $\delta _{ij}$ being the Kronecker delta and $\varvec{\epsilon }'_{ij}=\frac{1}{2} \left( \frac{\partial u'_i}{\partial x'_j} + \frac{\partial u'_j}{\partial x'_i}\right)$ the rate of strain tensor, $\mu '$ the dynamic viscosity and $p'$ the pressure . For simplicity of notation the decomposition of $\mu '$ into its vertical and horizontal components will be done at the end. Define

A13 $$\begin{aligned} {\mathbb {S}}'=\mu ' \left( \nabla ' {\textbf{u}}' +(\nabla ' {\textbf{u}}' )^T\right) =2\mu '\varvec{\epsilon }', \end{aligned}$$

and using (A12), (A13) and (A11), equation (A10) becomes

A14 $$\begin{aligned} \begin{aligned} \rho ' \left( \frac{\partial }{\partial t' }+{\textbf{u}}'\cdot \nabla '\right) {\textbf{u}}'&=\rho ' {\textbf{g}}' -\nabla ' p' -\frac{2}{3}\nabla '\left[ \mu ' \left( \nabla ' \cdot {\textbf{u}}' \right) \right] + \nabla ' \cdot \left( 2\mu ' \varvec{\epsilon }' \right) \\&=\rho ' {\textbf{g}}' -\nabla ' p' +\frac{2}{3}\nabla '\left[ \mu ' \left( \frac{1}{\rho '}\frac{D\rho '}{Dt'}\right) \right] + \nabla ' \cdot {\mathbb {S}}'.\\ \end{aligned} \end{aligned}$$

Equation (A14) is the general form of the Navier-Stokes equation.

It is common to assume $\mu '$ to be constant, hence obtaining

A15 $$\begin{aligned} \rho '\frac{D{\varvec{u}}'}{Dt'}=\rho ' {\textbf{g}}' -\nabla ' p' +\mu '\Delta '{\varvec{u}}' +\frac{1}{3}\mu ' \nabla '\left( \nabla '\cdot {\varvec{u}}'\right) \end{aligned}$$

which becomes

A16 $$\begin{aligned} \rho '\frac{D{\varvec{u}}'}{Dt'}=\rho ' {\textbf{g}}' -\nabla ' p' +\mu '\Delta '{\varvec{u}}' \end{aligned}$$

if the fluid is incompressible, namely $\nabla '\cdot {\varvec{u}}'=0,$ with $\Delta '$ being the Laplacian.

#### A.2.1 Classical Spherical Coordinates

A coordinate system suitable for the Earth, with the poles excluded, is the system of (right-handed) spherical coordinates $(\varphi , \theta , r')$: $r'$ is the distance from the Earth’s center, $\theta \in [-\nicefrac {\pi }{2},\nicefrac {\pi }{2}]$ is the angle of latitude and $\varphi \in [0,2\pi ]$ is the angle of longitude. The unit vectors in this system are $({\textbf{e}}_{\varphi }, {\textbf{e}}_{\theta }, {\textbf{e}}_r)$ and the corresponding velocity components are ${\varvec{u}}'=(u',v',w')$: ${\textbf{e}}_{\varphi }$ points from West to East, ${\textbf{e}}_{\theta }$ from South to North and ${\textbf{e}}_r$ points upwards. See Figure 6. The $(\varphi , \theta , r')$-system is associated with a point fixed on the sphere (other than at the two poles where the unit vectors are not well-defined) which is rotating about its polar axis (with constant angular speed $\Omega ' \approx 7.29\cdot 10^{-5} \,s^{-1}$).

The change of coordinates from spherical to Cartesian and vice-versa is given by

A17 $$\begin{aligned} \left\{ \begin{array}{ll} x'=r' \cos \theta \cos \varphi ,\\ y'=r' \cos \theta \sin \varphi ,\\ z'=r' \sin \theta , \end{array}\right. \qquad \left\{ \begin{array}{ll} \varphi = \tan ^{-1}\left( \frac{y'}{x'}\right) ,\\ \theta = \sin ^{-1}\left( \frac{z'}{\sqrt{x'^2+y'^2+z'^2}}\right) ,\\ r'=\sqrt{x'^2+y'^2+z'^2}. \end{array}\right. \end{aligned}$$

and the unit tangent vectors are

A18 $$\begin{aligned} \mathbf {e_{\varphi }}=\frac{\frac{\partial {\mathfrak {r}}}{\partial \varphi }}{\left| \frac{\partial {\mathfrak {r}}}{\partial \varphi }\right| },\qquad \mathbf {e_{\theta }}=\frac{\frac{\partial {\mathfrak {r}}}{\partial \theta }}{\left| \frac{\partial {\mathfrak {r}}}{\partial \theta }\right| },\qquad {\textbf{e}}_r=\frac{\frac{\partial {\mathfrak {r}}}{\partial r}}{\left| \frac{\partial {\mathfrak {r}}}{\partial r}\right| }, \end{aligned}$$

where ${\mathfrak {r}}=x(\varphi ,\theta , r) {\textbf{e}}_{1}+y(\varphi ,\theta , r) {\textbf{e}}_{2}+z(\varphi ,\theta , r) {\textbf{e}}_{3}$, leading to

A19 $$\begin{aligned} \left\{ \begin{array}{ll} \mathbf {e_{\varphi }}=-\sin \varphi \mathbf {e_1}+\cos \varphi \mathbf {e_2},\\ \mathbf {e_{\theta }}=-\cos \varphi \sin \theta \mathbf {e_1}-\sin \varphi \sin \theta \mathbf {e_2}+\cos \theta \mathbf {e_3},\\ {\textbf{e}}_{r}=\cos \varphi \cos \theta \mathbf {e_1}+\sin \varphi \cos \theta \mathbf {e_2}+\sin \theta \mathbf {e_3}, \end{array}\right. \end{aligned}$$

and

A20 $$\begin{aligned} \begin{aligned}&{\textsf{h}}_{\varphi }=\left| \frac{\partial {\mathfrak {r}}}{\partial \varphi }\right| =r' \cos \theta ,\qquad {\textsf{h}}_{\theta }=\left| \frac{\partial {\mathfrak {r}}}{\partial \theta }\right| =r' ,\qquad {\textsf{h}}_{r'}=\left| \frac{\partial {\mathfrak {r}}}{\partial r}\right| =1,\\&{\textsf{h}}:={\textsf{h}}_{\varphi }{\textsf{h}}_{\theta }{\textsf{h}}_{r'}=r'^2\cos \theta . \end{aligned} \end{aligned}$$

***The momentum equation in classical spherical coordinates***

Starting from (A14):

A21 $$\begin{aligned} \rho ' \left( \frac{\partial }{\partial t' }+{\textbf{u}}'\cdot \nabla '\right) {\textbf{u}}'=\rho ' {\textbf{g}}' -\nabla ' p' +\frac{2}{3}\nabla '\left[ \mu ' \left( \frac{1}{\rho '}\frac{D\rho '}{Dt'}\right) \right] + \nabla ' \cdot {\mathbb {S}}' \end{aligned}$$

we now compute the various terms in spherical coordinates.

The gradient in spherical coordinates with respect to the basis $(\mathbf {e_{\varphi }}, \mathbf {e_{\theta }},\mathbf {e_{r}})$ reads

A22 $$\begin{aligned} \nabla '= \left( \frac{1}{r'\cos \theta }\frac{\partial }{\partial \varphi },\ \frac{1}{r'}\frac{\partial }{\partial \theta } ,\ \frac{\partial }{\partial r'} \right) , \end{aligned}$$

while the advection term is (see Appendix A.1)

A23 $$\begin{aligned} ({\varvec{u}}'\cdot \nabla '){\varvec{u}}'=\left( \frac{u'}{r'\cos \theta }\frac{\partial }{\partial \varphi }+ \frac{v'}{r'}\frac{\partial }{\partial \theta } +w'\frac{\partial }{\partial r'}\right) \begin{pmatrix} u'\\ v'\\ w' \end{pmatrix} +\frac{1}{r'}\begin{pmatrix}-u'v'\tan \theta + u'w' \\ u'^2\tan \theta +v'w'\\ -u'^2-v'^2 \end{pmatrix}. \end{aligned}$$

The last term in the previous equation (A23) is called metric term and represents curvature effects of the Earth (see [32]).

The gradient of a vector is a tensor and can be computed as (see equation (A6) in Appendix A.1 ):

A24 $$\begin{aligned} \begin{aligned} (\nabla ' {\varvec{u}}')^T =&\nabla ' \otimes {\varvec{u}}'= \left( \mathbf {e_{\varphi }} \frac{1}{r'\cos \theta }\frac{\partial }{\partial \varphi }+ \mathbf {e_{\theta }} \frac{1}{r'}\frac{\partial }{\partial \theta }+\mathbf {e_{r}}\frac{\partial }{\partial r'}\right) \\&\otimes \left( u'\mathbf {e_{\varphi }} + v'\mathbf {e_{\theta }}+w'\mathbf {e_{r}} \right) \\ =&\frac{1}{r'\cos \theta }\frac{\partial u'}{\partial \varphi } {\textbf{e}}_{\varphi }\otimes {\textbf{e}}_{\varphi }+ \frac{u'}{r'\cos \theta } {\textbf{e}}_{\varphi }\otimes (\sin \theta {\textbf{e}}_{\theta }-\cos \theta {\textbf{e}}_r) \\&+ \frac{1}{r'\cos \theta }\frac{\partial v'}{\partial \varphi } {\textbf{e}}_{\varphi }\otimes {\textbf{e}}_{\theta }+ \frac{v'}{r'\cos \theta }{\textbf{e}}_{\varphi }\otimes (-\sin \theta {\textbf{e}}_{\varphi }) \\&+\frac{1}{r'\cos \theta }\frac{\partial w'}{\partial \varphi } {\textbf{e}}_{\varphi }\otimes {\textbf{e}}_r+ \frac{w'}{r'\cos \theta } {\textbf{e}}_{\varphi }\otimes (\cos \theta {\textbf{e}}_{\varphi }) \\&+ \frac{1}{r'}\frac{\partial u'}{\partial \theta } {\textbf{e}}_{\theta }\otimes {\textbf{e}}_{\varphi }+\frac{1}{r'}\frac{\partial v'}{\partial \theta } {\textbf{e}}_{\theta }\otimes {\textbf{e}}_{\theta }+\frac{v'}{r'}{\textbf{e}}_{\theta }\otimes (-{\textbf{e}}_r) \\&+\frac{1}{r'}\frac{\partial w'}{\partial \theta } {\textbf{e}}_{\theta }\otimes {\textbf{e}}_r+ \frac{w'}{r'}{\textbf{e}}_{\theta }\otimes {\textbf{e}}_{\theta }+ \frac{\partial u'}{\partial r'}{\textbf{e}}_r\otimes {\textbf{e}}_{\varphi }\\&+ \frac{\partial v'}{\partial r'} {\textbf{e}}_r\otimes {\textbf{e}}_{\theta }+ \frac{\partial w' }{\partial r'} {\textbf{e}}_r\otimes {\textbf{e}}_r. \end{aligned} \end{aligned}$$

Since ${\mathbb {S}}'=\mu ' (\nabla ' {\varvec{u}}'+(\nabla ' {\varvec{u}}')^T)$ is a symmetric tensor, the component of ${\mathbb {S}}'$ are^3^:

A25 $$\begin{aligned} \begin{aligned} {\mathbb {S}}'_{\varphi \varphi }=&2\mu '\left( \frac{1}{r'\cos \theta }\frac{\partial u'}{\partial \varphi } -\frac{v'}{r'}\tan \theta +\frac{w'}{r'}\right) ,\quad {\mathbb {S}}'_{\theta \theta }=2\mu '\left( \frac{1}{r'}\frac{\partial v'}{\partial \theta } + \frac{w'}{r'}\right) ,\\ {\mathbb {S}}'_{rr}=&2\mu '\left( \frac{\partial w' }{\partial r'} \right) ,\quad {\mathbb {S}}'_{\varphi \theta }={\mathbb {S}}'_{\theta \varphi }= \mu '\left( \frac{1}{r'}\frac{\partial u'}{\partial \theta } +\frac{u'}{r'}\tan \theta + \frac{1}{r'\cos \theta }\frac{\partial v'}{\partial \varphi }\right) , \\ {\mathbb {S}}'_{\varphi r}=&{\mathbb {S}}'_{r\varphi }=\mu '\left( -\frac{u'}{r'} +\frac{1}{r'\cos \theta }\frac{\partial w'}{\partial \varphi }+\frac{\partial u'}{\partial r'}\right) ,\\ {\mathbb {S}}'_{\theta r}=&{\mathbb {S}}'_{r\theta } = \mu '\left( -\frac{v'}{r'} +\frac{1}{r'}\frac{\partial w'}{\partial \theta } + \frac{\partial v'}{\partial r'}\right) . \\ \end{aligned} \end{aligned}$$

Note that $\nabla '\cdot {\mathbb {S}}'$ is a vector, and is computed applying (A9) to ${\mathbb {S}}'$ and using (A20).

Splitting into the three components we get:

$${\textbf{e}}_{\varphi }$$-component:

A26 $$\begin{aligned}  &   \frac{1}{r'^2\cos \theta }\left[ \frac{\partial }{\partial \varphi }\left( r'{\mathbb {S}}'_{\varphi \varphi }\right) + \frac{\partial }{\partial \theta }\left( r'\cos \theta {\mathbb {S}}'_{\theta \varphi }\right) + \frac{\partial }{\partial r'}\left( r'^2\cos \theta {\mathbb {S}}'_{r\varphi }\right) \right] \nonumber \\  &   \quad +\frac{{\mathbb {S}}'_{\varphi \theta }(-r'\sin \theta )}{r'^2\cos \theta }+\frac{{\mathbb {S}}'_{r\varphi }\cos \theta }{r'\cos \theta }\nonumber \\  &   \quad =\frac{1}{r'\cos \theta }\frac{\partial {\mathbb {S}}'_{\varphi \varphi }}{\partial \varphi } + \frac{1}{r'\cos \theta }\frac{\partial }{\partial \theta }{(\cos \theta {\mathbb {S}}'_{\theta \varphi })} + \frac{1}{r'^2}\frac{\partial }{\partial r'}{(r'^2{\mathbb {S}}'_{r\varphi })} \nonumber \\  &   \quad +\frac{{\mathbb {S}}'_{r\varphi }-{\mathbb {S}}'_{\varphi \theta }\tan \theta }{r'} \nonumber \\  &   \quad =\underbrace{\frac{\mu '}{r'^2\cos ^2\theta }\frac{\partial ^2 u'}{\partial \varphi ^2} -\frac{\mu ' }{r'^2}\tan \theta \frac{\partial u'}{\partial \theta } +\frac{\mu ' }{r'^2}\frac{\partial ^2 u'}{\partial \theta ^2}+2\frac{\mu '}{r'}\frac{\partial u}{\partial r'}+\mu '\frac{\partial ^2 u'}{\partial r'^2}}_{=\mathbin \bigtriangleup '_{\mu }u'}\nonumber \\  &   \quad +\left[ -\frac{2\mu '}{r'^2}\frac{\tan \theta }{\cos \theta }\frac{\partial v'}{\partial \varphi }+\frac{2\mu '}{r'^2\cos \theta }\frac{\partial w'}{\partial \varphi } -\frac{\mu '}{r'^2}u'-\frac{\mu ' \tan ^2\theta }{r'^2}u'\right] \nonumber \\  &   \quad +\left[ \frac{d\mu '}{d r'}\left( \frac{\partial u'}{\partial r'}-\frac{u'}{r'}+\frac{1}{r'\cos \theta }\frac{\partial w'}{\partial \varphi }\right) \right] +\frac{\mu '}{r'^2\cos ^2\theta }\frac{\partial ^2 u'}{\partial \varphi ^2}+ \frac{\mu '}{r'^2\cos \theta }\frac{\partial ^2 v'}{\partial \theta \partial \varphi }\nonumber \\  &   \quad +\frac{\mu '}{r'^2\cos \theta }\frac{\partial w'}{\partial \varphi }+\frac{\mu '}{r'\cos \theta }\frac{\partial ^2 w'}{\partial r' \partial \varphi }+\frac{\mu '}{r'^2\cos \theta }\frac{\partial w'}{\partial \varphi }- \frac{\mu ' \tan \theta }{r'^2\cos \theta }\frac{\partial v'}{\partial \varphi }, \end{aligned}$$

$${\textbf{e}}_{\theta }$$-component:

A27 $$\begin{aligned} \begin{aligned}&\frac{1}{r'^2\cos \theta }\left[ \frac{\partial }{\partial \varphi }\left( r'{\mathbb {S}}'_{\theta \varphi }\right) + \frac{\partial }{\partial \theta }\left( r'\cos \theta {\mathbb {S}}'_{\theta \theta }\right) + \frac{\partial }{\partial r'}\left( r'^2\cos \theta {\mathbb {S}}'_{\theta r}\right) \right] \\&\quad +\frac{{\mathbb {S}}'_{\theta r}}{r'}+\frac{{\mathbb {S}}'_{\varphi \varphi }r'\sin \theta }{r'^2\cos \theta }\\&=\frac{1}{r'\cos \theta }\frac{\partial {\mathbb {S}}'_{\theta \varphi }}{\partial \varphi } + \frac{1}{r'\cos \theta }\frac{\partial }{\partial \theta } {(\cos \theta {\mathbb {S}}'_{\theta \theta })}+ \frac{1}{r'^2}\frac{\partial }{\partial r'}{(r'^2{\mathbb {S}}'_{\theta r})} \\&\quad +\frac{{\mathbb {S}}'_{\theta r}+{\mathbb {S}}'_{\varphi \varphi }\tan \theta }{r'} \\&=\underbrace{\frac{\mu '}{r'^2\cos ^2\theta }\frac{\partial ^2 v'}{\partial \varphi ^2} -\frac{\mu ' }{r'^2}\tan \theta \frac{\partial v'}{\partial \theta } +\frac{\mu ' }{r'^2}\frac{\partial ^2 v'}{\partial \theta ^2}+2\frac{\mu '}{r'}\frac{\partial v'}{\partial r'}+\mu '\frac{\partial ^2 v'}{\partial r'^2}}_{=\mathbin \bigtriangleup '_{\mu }v'} \\&\quad +\left[ \frac{2\mu '}{r'^2}\frac{\partial w'}{\partial \theta } -\frac{\mu ' v'}{r'^2}+\frac{2\mu '\tan \theta }{r'^2\cos \theta }\frac{\partial u'}{\partial \varphi }-\frac{\mu '\tan ^2\theta }{r'^2}v'\right] \\&\quad +\left[ \frac{d\mu '}{dr'}\left( \frac{\partial v'}{\partial r'}-\frac{v'}{r'}+\frac{1}{r'}\frac{\partial w'}{\partial \theta }\right) \right] +\frac{\mu '\tan \theta }{r'^2\cos \theta }\frac{\partial u'}{\partial \varphi }+\frac{\mu '}{r'^2\cos \theta }\frac{\partial ^2 u'}{\partial \varphi \partial \theta } \\&\quad + \frac{\mu '}{r'^2}\frac{\partial ^2v'}{\partial \theta ^2}-\frac{\mu '}{r'^2}\tan \theta \frac{\partial v'}{\partial \theta }-\frac{\mu '}{r'^2}v'+2\frac{\mu '}{r'^2}\frac{\partial w'}{\partial \theta }+\frac{\mu '}{r'}\frac{\partial ^2 w' }{\partial r' \partial \theta }-\frac{\mu '\tan ^2\theta }{r'^2}v', \end{aligned} \end{aligned}$$

$${\textbf{e}}_r$$-component:

A28 $$\begin{aligned}  &   \frac{1}{r'^2\cos \theta }\left[ \frac{\partial }{\partial \varphi }\left( r'{\mathbb {S}}'_{r\varphi }\right) + \frac{\partial }{\partial \theta }\left( r'\cos \theta {\mathbb {S}}'_{r\varphi }\right) + \frac{\partial }{\partial r'}\left( r'^2\cos \theta {\mathbb {S}}'_{rr}\right) \right] \nonumber \\  &   \quad -\frac{{\mathbb {S}}'_{\varphi \varphi }\cos \theta }{r'\cos \theta }-\frac{{\mathbb {S}}'_{\theta \theta }}{r'}\nonumber \\  &   \quad =\frac{1}{r'\cos \theta }\frac{\partial {\mathbb {S}}'_{r\varphi }}{\partial \varphi } + \frac{1}{r'\cos \theta }\frac{\partial }{\partial \theta }{(\cos \theta {\mathbb {S}}'_{r\theta })} + \frac{1}{r'^2}\frac{\partial }{\partial r'}{(r'^2{\mathbb {S}}'_{rr})} \nonumber \\  &   \quad -\frac{{\mathbb {S}}'_{\varphi \varphi }+{\mathbb {S}}'_{\theta \theta }}{r'} \nonumber \\  &   \quad =\underbrace{\frac{\mu '}{r'^2\cos ^2\theta }\frac{\partial ^2 w'}{\partial \varphi ^2} -\frac{\mu ' }{r'^2}\tan \theta \frac{\partial w'}{\partial \theta } +\frac{\mu ' }{r'^2}\frac{\partial ^2 w'}{\partial \theta ^2}+2\frac{\mu '}{r'}\frac{\partial w'}{\partial r'}+\mu '\frac{\partial ^2 w'}{\partial r'^2}}_{=\mathbin \bigtriangleup '_{\mu }w'} \nonumber \\  &   \quad +\left[ -\frac{2\mu '}{r'^2\cos \theta }\frac{\partial u'}{\partial \varphi } +\frac{2\mu '}{r'^2}\tan \theta v' -2\frac{\mu '}{r'^2}w'-\frac{2\mu '}{r'^2}\frac{\partial v'}{\partial \theta }\right] +\left[ 2\frac{d\mu '}{dr'}\frac{\partial w'}{\partial r'}\right] \nonumber \\  &   \quad -\frac{\mu '}{r'^2\cos \theta }\frac{\partial u'}{\partial \varphi }+\frac{\mu '}{r'\cos \theta }\frac{\partial ^2 u'}{\partial \varphi \partial r'}+\frac{\mu '}{r'^2}\tan \theta v' -\frac{\mu '}{r'}\tan \theta \frac{\partial v'}{\partial r'}-\frac{\mu '}{r'^2} \frac{\partial v'}{\partial \theta } \nonumber \\  &   \quad +\frac{\mu '}{r'} \frac{\partial ^2 v'}{\partial \theta \partial r'}+\frac{2\mu '}{r'}\frac{\partial w'}{\partial r'} +\mu '\frac{\partial ^2 w'}{\partial r'^2} -2\frac{\mu '}{r'^2}w', \end{aligned}$$

where

A29 $$\begin{aligned} \mathbin \bigtriangleup '_{\mu }:= \left[ \mu ' \left( \frac{\partial ^2}{\partial r'^2}+ \frac{2}{r'}\frac{\partial }{\partial r'}\right) + \frac{\mu '}{r'^2}\left( \frac{1}{ \cos ^2 \theta } \frac{\partial ^2 }{\partial \varphi ^2}+\frac{\partial ^2}{\partial \theta ^2} - \tan \theta \frac{\partial }{\partial \theta }\right) \right] . \end{aligned}$$

Now, observe that, computing $\nabla '(\mu ' \nabla '\cdot {\varvec{u}}')$ and recalling that we are considering $\mu '=\mu '(r)$, it follows:

A30 $$\begin{aligned}  &   \begin{aligned}&\frac{1}{r'\cos \theta }\frac{\partial }{\partial \varphi }\left[ \mu '\left( \frac{1}{r' \cos \theta } \frac{\partial u'}{\partial \varphi } + \frac{1}{r' \cos \theta } \frac{\partial }{\partial \theta } \left( v' \cos \theta \right) + \frac{1}{r'^2} \frac{\partial }{\partial r'} \left( r'^2 w' \right) \right) \right] \\&=\frac{\mu '}{r'^2 \cos ^2\theta } \frac{\partial ^2 u'}{\partial \varphi ^2}+\frac{\mu '}{r'^2 \cos \theta } \frac{\partial ^2 v'}{\partial \varphi \partial \theta } - \frac{\mu '}{r'^2 \cos \theta } \tan \theta \frac{\partial v'}{\partial \varphi } \\&\quad +\frac{\mu '}{r'\cos \theta }\frac{\partial ^2 w'}{\partial \varphi \partial r'}+ \frac{2\mu '}{r'^2\cos \theta } \frac{\partial w'}{\partial \varphi }, \end{aligned} \end{aligned}$$

A31 $$\begin{aligned}  &   \begin{aligned}&\frac{1}{r'}\frac{\partial }{\partial \theta }\left[ \mu '\left( \frac{1}{r' \cos \theta } \frac{\partial u'}{\partial \varphi } + \frac{1}{r' \cos \theta } \frac{\partial }{\partial \theta } \left( v' \cos \theta \right) + \frac{1}{r'^2} \frac{\partial }{\partial r'} \left( r'^2 w' \right) \right) \right] \\&=\frac{\mu '\tan \theta }{r'^2\cos \theta }\frac{\partial u'}{\partial \varphi }+\frac{\mu '}{r'^2\cos \theta }\frac{\partial ^2 u'}{\partial \varphi \partial \theta } + \frac{\mu '}{r'^2}\frac{\partial ^2v'}{\partial \theta ^2}-\frac{\mu '}{r'^2}\tan \theta \frac{\partial v'}{\partial \theta }\\&\quad -\frac{\mu '}{r'^2\cos ^2\theta }v'+2\frac{\mu '}{r'^2}\frac{\partial w'}{\partial \theta }+\frac{\mu '}{r'}\frac{\partial ^2 w' }{\partial r' \partial \theta }, \end{aligned} \end{aligned}$$

A32 $$\begin{aligned}  &   \begin{aligned}&\frac{\partial }{\partial r'}\left[ \mu '\left( \frac{1}{r' \cos \theta } \frac{\partial u'}{\partial \varphi } + \frac{1}{r' \cos \theta } \frac{\partial }{\partial \theta } \left( v' \cos \theta \right) + \frac{1}{r'^2} \frac{\partial }{\partial r'} \left( r'^2 w' \right) \right) \right] \\&=\left( \frac{d\mu '}{d r'}\right) \left( \frac{1}{r' \cos \theta } \frac{\partial u'}{\partial \varphi } + \frac{1}{r' \cos \theta } \frac{\partial }{\partial \theta } \left( v' \cos \theta \right) + \frac{1}{r'^2} \frac{\partial }{\partial r'} \left( r'^2 w' \right) \right) \\&\quad +\mu '\frac{\partial }{\partial r'}\left[ \left( \frac{1}{r' \cos \theta } \frac{\partial u'}{\partial \varphi } + \frac{1}{r' \cos \theta } \frac{\partial }{\partial \theta } \left( v' \cos \theta \right) + \frac{1}{r'^2} \frac{\partial }{\partial r'} \left( r'^2 w' \right) \right) \right] \\&=\left( \frac{d\mu '}{d r'}\right) \nabla '\cdot {\varvec{u}}'-\frac{\mu '}{r'^2\cos \theta }\frac{\partial u'}{\partial \varphi }+\frac{\mu '}{r'\cos \theta }\frac{\partial ^2 u'}{\partial \varphi \partial r'}+\mu '\frac{\partial ^2 w'}{\partial r'^2} +\frac{2\mu '}{r'}\frac{\partial w'}{\partial r'} \\&\quad -2\frac{\mu '}{r'^2}w'+\frac{\mu '}{r'^2}\tan \theta v'-\frac{\mu '}{r'}\tan \theta \frac{\partial v'}{\partial r'}-\frac{\mu '}{r'^2} \frac{\partial v'}{\partial \theta } +\frac{\mu '}{r'} \frac{\partial ^2 v'}{\partial \theta \partial r'}. \end{aligned} \end{aligned}$$

Comparing (A30), (A31), (A32) with the last terms of (A26), (A27), (A28), using the continuity equation (A11) to write $\mu ' \nabla ' \cdot {\textbf{u}}' = -\frac{\mu '}{\rho '} \frac{D \rho '}{D t'}$, and recalling that the divergence of the vector field ${\varvec{u}}'=(u',v',w')$ in spherical coordinates $(\varphi ,\theta , r')$ is

A33 $$\begin{aligned} \begin{aligned} \nabla '\cdot {\varvec{u}}'&=\frac{1}{r' \cos \theta } \frac{\partial u'}{\partial \varphi } + \frac{1}{r' \cos \theta } \frac{\partial }{\partial \theta } \left( v' \cos \theta \right) + \frac{1}{r'^2} \frac{\partial }{\partial r'} \left( r'^2 w'\right) \\&= \frac{1}{r' \cos \theta } \frac{\partial u'}{\partial \varphi } + \frac{1}{r'} \frac{\partial v'}{\partial \theta } - \frac{v'}{r'} \tan \theta + \frac{\partial w'}{\partial r'} + \frac{2}{r'} w', \end{aligned} \end{aligned}$$

the momentum equation in spherical coordinates becomes

A34 $$\begin{aligned}&\quad \rho '\frac{D}{Dt'}\begin{pmatrix} u' \\ v' \\ w'\\ \end{pmatrix} +\frac{\rho '}{r'}\begin{pmatrix}-u'v'\tan \theta + u'w' \\ u'^2\tan \theta +v'w'\\ -u'^2-v'^2 \end{pmatrix}\nonumber \\&\quad +2\rho '\Omega '\begin{pmatrix} -v'\sin \theta +w'\cos \theta \\ u'\sin \theta \\ -u'\cos \theta \end{pmatrix}+\rho ' r' \Omega '^2\begin{pmatrix} 0\\ \sin \theta \cos \theta \\ -\cos ^2\theta \end{pmatrix} \nonumber \\&=-\nabla ' p' +\rho '\begin{pmatrix} 0\\ 0\\ -g'\frac{R'^2}{ r'^2}\\ \end{pmatrix}+ \Delta '_{\mu } \begin{pmatrix} u'\\ v'\\ w' \end{pmatrix}-\frac{1}{3}\begin{pmatrix} \frac{1}{r'\cos \theta }\frac{\partial }{\partial \varphi }\left( \frac{\mu '_H}{\rho '} \frac{D\rho '}{D t'}\right) \\ \frac{1}{r'}\frac{\partial }{\partial \theta }\left( \frac{\mu '_H}{\rho '} \frac{D\rho '}{D t'}\right) \\ \frac{\partial }{\partial r'}\left( \frac{\mu '_V}{\rho '} \frac{D\rho '}{D t'}\right) \end{pmatrix} \nonumber \\&\quad -\frac{1}{r'^2\cos ^2\theta }\begin{pmatrix} \mu '_H u\\ \mu '_H v\\ 2\mu '_V(w'\cos ^2\theta -v'\sin \theta \cos \theta ) \end{pmatrix}+\frac{2\mu '_H}{r'^2}\frac{\partial }{\partial \theta }\begin{pmatrix} 0\\ w'\\ -v' \end{pmatrix} \nonumber \\&\quad +\frac{2\mu '_H}{r'^2\cos \theta }\frac{\partial }{\partial \phi }\begin{pmatrix} w'-v'\tan \theta \\ u'\tan \theta \\ -u' \end{pmatrix}+ \frac{d \mu '_V}{dr'}r' \begin{pmatrix} \frac{\partial }{\partial r'}\left( \frac{u'}{r'}\right) \\ \frac{\partial }{\partial r'}\left( \frac{v'}{r'}\right) \\ {0} \end{pmatrix}\nonumber \\&\quad +\frac{d \mu '_H}{d r'} \begin{pmatrix} \frac{1}{r'\cos \theta }\frac{\partial {w'}}{\partial \phi }\\ \frac{1}{r'} \frac{\partial {w'}}{\partial \theta }\\ {0} \end{pmatrix}+ \frac{d \mu '_V}{dr'} \begin{pmatrix} 0\\ 0\\ 2 \frac{\partial w'}{\partial r'} \end{pmatrix}\nonumber \\&\quad +\frac{d \mu '_V}{dr'}\begin{pmatrix} 0\\ 0\\ \frac{1}{r' \cos \theta } \frac{\partial u'}{\partial \varphi } + \frac{1}{r' \cos \theta } \frac{\partial }{\partial \theta } \left( v' \cos \theta \right) + \frac{1}{r'^2} \frac{\partial }{\partial r'} \left( r'^2 w' \right) \end{pmatrix}, \end{aligned}$$

where

A35 $$\begin{aligned} \begin{aligned} \nabla '&= \left( \frac{1}{r'\cos \theta }\frac{\partial }{\partial \varphi },\ \frac{1}{r'}\frac{\partial }{\partial \theta } ,\ \frac{\partial }{\partial r'} \right) ,\\ \frac{D}{Dt'}&=\frac{\partial }{\partial t'}+ \frac{u'}{r' \cos \theta } \frac{\partial }{\partial \varphi } + \frac{v'}{r'} \frac{\partial }{\partial \theta } + w'\frac{\partial }{\partial r'}, \\ \Delta '_{\mu }&=\mu '_V \left( \frac{\partial ^2}{\partial r'^2}+ \frac{2}{r'}\frac{\partial }{\partial r'}\right) + \frac{\mu '_H}{r'^2}\left( \frac{1}{ \cos ^2 \theta } \frac{\partial ^2 }{\partial \varphi ^2}+\frac{\partial ^2}{\partial \theta ^2} - \tan \theta \frac{\partial }{\partial \theta }\right) , \end{aligned} \end{aligned}$$

$R' \approx 6371\ km$ is the Earth’s radius, $g' \approx 9.81 \ m\, s^{-2}$ is the average acceleration of gravity at the surface of the Earth, and we have now made the distinction between $\mu '_V$ (vertical) and $\mu '_H$ (horizontal).

The quantities $\mu '_V$ and $\mu '_H$ are the so-called dynamics (effective) viscosities, the sum of the molecular and eddy (related to Reynolds stresses) viscosities [28]. The eddy viscosity is several orders of magnitude bigger than the molecular viscosity and is a model for the so-called closure problem in turbulence modeling. Eddy viscosity models are based on two key principles: the Boussinesq hypothesis (stating that turbulent fluctuations dissipate mean flow energy), and the eddy viscosity hypothesis (stating that Reynolds (shear) stresses are proportional to the mean velocity gradient). See [33] for a summary of recent advances in the mathematical foundations of eddy viscosity models of turbulence.

For oceans, the kinematic eddy viscosity $A'_V:=\frac{\mu '_V}{\rho '}$ varies between $10^{-4}\ m^2\, s^{-1}$ and $10^{-1}\ m^2\, s^{-1}$, while $A'_H:=\frac{\mu '_H}{\rho '}$ ranges between $10\ m^2\, s^{-1}$ and $10^{4}\ m^2\, s^{-1}$.

In case of atmospheric flows, values of $A'_V$ are $10\ m^2\, s^{-1}$ near the Earth’s surface are considerably lower in the atmosphere, while $A'_H$ has order of $10\ m^2\, s^{-1}$ [28].

As the eddy viscosity reflects “the intensity of the whirling agitation” (see [34]) and under-ice motion is less turbulent than the one with a free-surface, we may expect to have $A'_H$ values even smaller than $10\ m^2\, s^{-1}$ in this case.

Moreover, in the left-hand side of (A34) we have also added the terms $2\varvec{\Omega }'\times {\varvec{u}}'$ (Coriolis acceleration) and $\varvec{\Omega }'\times (\varvec{\Omega }'\times {\varvec{r}}')$ (centrifugal acceleration, ${\varvec{r}}'$ being the position vector) due to the Earth’s rotation around its vertical axis ${\textbf{e}}_3$ with angular with angular velocity $\Omega ' \approx 7.29\cdot 10^{-5} \, rad\,s^{-1}$, $\varvec{\Omega }'=\Omega '{\textbf{e}}_3$.

Equation (A34) is consistent with the standard one in Cartesian coordinates (A15).

***The continuity equation in classical spherical coordinates*** The continuity equation $\frac{D\rho '}{D t'} +\rho ' \nabla '\cdot \mathbf {u'}=0$ in spherical coordinates, using the above mentioned formula for the divergence, reads

A36 $$\begin{aligned} \frac{D\rho '}{D t'} +\rho '\left[ \frac{1}{r' \cos \theta } \frac{\partial u'}{\partial \varphi } + \frac{1}{r' \cos \theta } \frac{\partial }{\partial \theta } \left( v' \cos \theta \right) + \frac{1}{r'^2} \frac{\partial }{\partial r'} \left( r'^2 w' \right) \right] =0. \end{aligned}$$

#### A.2.2 Rotated Spherical Coordinates

Classical spherical coordinates present two polar singularities, where longitude is undefined at the North and South Poles. This issue reflects the fundamental impossibility of defining global coordinates on a sphere: singularities, where the coordinate system either fails or degenerates, are inevitable. According to the Poincaré–Hopf index theorem, continuous vector fields free of singularities can exist only on manifolds with a zero Euler characteristic. Since the sphere has an Euler characteristic of 2, there cannot exist a continuous, nonvanishing tangent vector field on it. This result is commonly referred to as the “hairy ball theorem” (see [35]).

The convergence of meridians at the poles illustrates this limitation in spherical coordinates. However, the problem can be resolved by rotating the coordinate system so that both singularities are relocated to the Equator. This adjustment enables a smooth parameterization for the entire region north (or south) of the Equator. In this section we review the construction of this rotated spherical coordinates developed in [4]. See also [9].

Start by considering the standard Cartesian coordinate system $({\textbf{e}}_1, {\textbf{e}}_2, {\textbf{e}}_3)$ positioned at the center of the Earth, pointing in the direction $\vec {OZ}$ of Null Island, $\vec {OE}$ East, $\vec {ON}$ North, respectively. See Figure 7.

Now, define a new Cartesian coordinate system $({\textbf{e}}_1^{\dag }, {\textbf{e}}_2^{\dag }, {\textbf{e}}_3^{\dag })$ by permuting cyclically the first three Cartesian axes

A37 $$\begin{aligned} {\textbf{e}}_1^{\dag }={\textbf{e}}_{3},\quad {\textbf{e}}_2^{\dag }={\textbf{e}}_{1}, \quad {\textbf{e}}_3^{\dag }={\textbf{e}}_{2}. \end{aligned}$$

In terms of the associated azimuthal $\theta ^{\dag }\in [-\frac{\pi }{2},\frac{\pi }{2}]$ and meridional $\varphi ^{\dag }\in [0,2\pi )$ angles, the coordinates of a point *P* on the Earth are

A38 $$\begin{aligned} r'\cos \theta ^{\dag }\cos \varphi ^{\dag }{\textbf{e}}_{1}^{\dag }+r'\cos \theta ^{\dag }\sin \varphi ^{\dag }{\textbf{e}}_{2}^{\dag }+r'\sin \theta ^{\dag }{\textbf{e}}_{3}^{\dag }, \end{aligned}$$

while in the standard system they are

A39 $$\begin{aligned} r'\cos \theta \cos \varphi {\textbf{e}}_{1}+r'\cos \theta \sin \varphi {\textbf{e}}_{2}+r'\sin \theta {\textbf{e}}_{3}. \end{aligned}$$

Therefore, using (A37), it follows that

A40 $$\begin{aligned} \left\{ \begin{aligned}&\cos \theta ^{\dag }\cos \varphi ^{\dag }=\cos \theta \sin \varphi ,\\&\cos \theta ^{\dag }\sin \varphi ^{\dag }=\sin \theta ,\\&\sin \theta ^{\dag }=\cos \theta \cos \varphi . \end{aligned}\right. \end{aligned}$$

In this new coordinate system the North Pole has coordinates $\varphi ^{\dag }=\frac{\pi }{2},\ \theta ^{\dag }=0$, and the solution of the system (A40) for the Arctic Ocean is

A41 $$\begin{aligned} \left\{ \begin{aligned}&\varphi ^{\dag }=\cot ^{-1}(\sin \varphi \cot \theta ) \in (0,\pi ),\\&\theta ^{\dag }=\sin ^{-1}(\cos \varphi \cos \theta ) \in \left( -\frac{\pi }{2},\frac{\pi }{2}\right) , \end{aligned}\right. \end{aligned}$$

The unit vectors in the rotated spherical coordinates $(\varphi ^{\dag }, \theta ^{\dag }, r')$ are $({\textbf{e}}_{\varphi }^{\dag }, {\textbf{e}}_{\theta }^{\dag }, {\textbf{e}}_r)$, given by

A42 $$\begin{aligned} \left\{ \begin{array}{ll} {\textbf{e}}_{\varphi }^{\dag }=-\sin \varphi ^{\dag } {\textbf{e}}_{1}^{\dag }+\cos \varphi ^{\dag } {\textbf{e}}_{2}^{\dag },\\ {\textbf{e}}_{\theta }^{\dag }=-\cos \varphi ^{\dag }\sin \theta ^{\dag } {\textbf{e}}_{1}^{\dag }-\sin \varphi ^{\dag }\sin \theta ^{\dag } {\textbf{e}}_{2}^{\dag }+\cos \theta ^{\dag } {\textbf{e}}_{3}^{\dag },\\ {\textbf{e}}_r^{\dag }=\cos \varphi ^{\dag }\cos \theta ^{\dag } {\textbf{e}}_{1}^{\dag }+\sin \varphi ^{\dag }\cos \theta ^{\dag } {\textbf{e}}_{2}^{\dag }+\sin \theta ^{\dag } {\textbf{e}}_{3}^{\dag }, \end{array}\right. \end{aligned}$$

and the corresponding velocity components are $(u'_{\dag }, v'_{\dag }, w'_{\dag })$.

In the new rotated spherical coordinates the momentum equations are

A43 $$\begin{aligned}  &   \rho ' \frac{D}{D t'}\begin{pmatrix} u'_{\dag } \\ v'_{\dag }\\ w'_{\dag }\\ \end{pmatrix}+\frac{\rho '}{r'}\begin{pmatrix}-u'_{\dag }v'_{\dag }\tan \theta ^{\dag } + u'_{\dag }w'_{\dag }\\ u'^2_{\dag }\tan \theta ^{\dag }+v'_{\dag }w'_{\dag }\\ -u'^2_{\dag }-v'^2_{\dag } \end{pmatrix}\nonumber \\  &   \qquad +2\rho '\Omega '\begin{pmatrix} -v'_{\dag }\sin \varphi ^{\dag }\cos \theta ^{\dag }-w'_{\dag }\sin \varphi ^{\dag }\sin \theta ^{\dag }\\ u'_{\dag }\sin \varphi ^{\dag }\cos \theta ^{\dag }-w'_{\dag }\cos \varphi ^{\dag }\\ u'_{\dag } \sin \varphi ^{\dag }\sin \theta ^{\dag } +v'_{\dag } \cos \varphi ^{\dag } \end{pmatrix}\nonumber \\  &   \qquad +\rho ' r' \Omega '^2\begin{pmatrix} \sin \varphi ^{\dag }\cos \varphi ^{\dag }\cos \theta ^{\dag }\\ -\sin ^2\varphi ^{\dag } \sin \theta ^{\dag }\cos \theta ^{\dag }\\ -\cos ^2\varphi ^{\dag }\cos ^2\theta ^{\dag } -\sin ^2\theta ^{\dag } \end{pmatrix} \nonumber \\  &   \quad =-\nabla 'p' +\rho \begin{pmatrix} 0\\ 0\\ -g'\frac{R'^2}{ r'^2}\\ \end{pmatrix} +\Delta '_{\mu } \begin{pmatrix} u'_{\dag }\\ v'_{\dag }\\ w'_{\dag } \end{pmatrix}-\frac{1}{3}\begin{pmatrix} \frac{1}{r'\cos \theta ^{\dag }}\frac{\partial }{\partial \varphi ^{\dag }}\left( \frac{\mu '_H}{\rho '} \frac{D\rho '}{D t'}\right) \\ \frac{1}{r'}\frac{\partial }{\partial \theta ^{\dag }}\left( \frac{\mu '_H}{\rho '} \frac{D\rho '}{D t'}\right) \\ \frac{\partial }{\partial r'}\left( \frac{\mu '_V}{\rho '} \frac{D\rho '}{D t'}\right) \end{pmatrix}\nonumber \\  &   \qquad -\frac{1}{r'^2\cos ^2\theta ^{\dag }}\begin{pmatrix} \mu '_H u'_{\dag }\\ \mu '_H v'_{\dag }\\ 2\mu '_V(w'_{\dag }\cos ^2\theta ^{\dag }-v'_{\dag }\sin \theta ^{\dag }\cos \theta ^{\dag }) \end{pmatrix}\nonumber \\  &   \qquad + \frac{2\mu '_H}{r'^2}\frac{\partial }{\partial \theta ^{\dag }}\begin{pmatrix} 0\\ w'_{\dag }\\ -v'_{\dag } \end{pmatrix}+\frac{2\mu '_H}{r'^2\cos \theta ^{\dag }}\frac{\partial }{\partial \varphi ^{\dag }}\begin{pmatrix} w'_{\dag }-v'_{\dag }\tan \theta ^{\dag }\\ u'_{\dag }\tan \theta ^{\dag }\\ -u'_{\dag } \end{pmatrix} \nonumber \\  &   \qquad +\frac{d \mu '_V}{dr'}r' \begin{pmatrix} \frac{\partial }{\partial r'}\left( \frac{u'_{\dag }}{r'}\right) \\ \frac{\partial }{\partial r'}\left( \frac{v'_{\dag }}{r'}\right) \\ {0} \end{pmatrix}+\frac{d \mu '_H}{d r'} \begin{pmatrix} \frac{1}{r'\cos \theta }\frac{\partial {w'_{\dag }}}{\partial \varphi ^{\dag }}\\ \frac{1}{r'} \frac{\partial {w'_{\dag }}}{\partial \theta ^{\dag }}\\ {0} \end{pmatrix}+ \frac{d \mu '_V}{dr'} \begin{pmatrix} 0\\ 0\\ 2 \frac{\partial w'_{\dag }}{\partial r'} \end{pmatrix}\nonumber \\  &   \qquad +\frac{d \mu '_V}{dr'}\begin{pmatrix} 0\\ 0\\ \frac{1}{r' \cos \theta ^{\dag }} \frac{\partial u'_{\dag }}{\partial \varphi ^{\dag }} + \frac{1}{r' \cos \theta ^{\dag }} \frac{\partial }{\partial \theta ^{\dag }} \left( v'_{\dag } \cos \theta ^{\dag } \right) + \frac{1}{r'^2} \frac{\partial }{\partial r'} \left( r'^2 w'_{\dag } \right) \end{pmatrix}, \end{aligned}$$

where

A44 $$\begin{aligned} \begin{aligned} \nabla '&= \left( \frac{1}{r'\cos \theta ^{\dag }}\frac{\partial }{\partial \varphi ^{\dag }},\ \frac{1}{r'}\frac{\partial }{\partial \theta ^{\dag }} ,\ \frac{\partial }{\partial r'} \right) ,\\ \frac{D}{Dt'}&=\frac{\partial }{\partial t'}+ \frac{u'_{\dag }}{r' \cos \theta ^{\dag }} \frac{\partial }{\partial \varphi ^{\dag }} + \frac{v'_{\dag }}{r'} \frac{\partial }{\partial \theta ^{\dag }} + w'_{\dag }\frac{\partial }{\partial r'}, \\ \Delta '_{\mu }&=\mu '_V \left( \frac{\partial ^2}{\partial r'^2}+ \frac{2}{r'}\frac{\partial }{\partial r'}\right) + \frac{\mu '_H}{r'^2}\left( \frac{1}{ \cos ^2 \theta ^{\dag }} \frac{\partial ^2 }{\partial \varphi ^{\dag 2}}+\frac{\partial ^2}{\partial \theta ^{\dag 2}} - \tan \theta ^{\dag }\frac{\partial }{\partial \theta ^{\dag }}\right) , \end{aligned} \end{aligned}$$

while the continuity equation is

A45 $$\begin{aligned} \frac{D'\rho }{Dt'}+\rho '\left[ \frac{1}{r' \cos \theta ^{\dag }} \frac{\partial u'_{\dag }}{\partial \varphi ^{\dag }} + \frac{1}{r' \cos \theta ^{\dag }} \frac{\partial }{\partial \theta ^{\dag }} \left( v'_{\dag } \cos \theta ^{\dag } \right) + \frac{1}{r'^2} \frac{\partial }{\partial r'} \left( r'^2 w'_{\dag }\right) \right] =0. \end{aligned}$$

The Navier-Stokes equations in the new spherical coordinate system (A43) look very similar to the ones in the classical spherical system (A34), except for two points: in (A43) the Coriolis acceleration and the centrifugal acceleration terms are now given by, respectively,

A46 $$\begin{aligned} \begin{aligned}&2\mathbf {\Omega }'\times {\textbf{u}}'=2\Omega '\begin{pmatrix} -v'_{\dag }\sin \varphi ^{\dag }\cos \theta ^{\dag }-w'_{\dag }\sin \varphi ^{\dag }\sin \theta ^{\dag }\\ u'_{\dag }\sin \varphi ^{\dag }\cos \theta ^{\dag }-w'_{\dag }\cos \varphi ^{\dag }\\ u'_{\dag } \sin \varphi ^{\dag }\sin \theta ^{\dag } +v'_{\dag } \cos \varphi ^{\dag } \end{pmatrix},\\&\mathbf {\Omega }'\times (\mathbf {\Omega }'\times {\textbf{r}}')= r' \Omega '^2\begin{pmatrix} \sin \varphi ^{\dag }\cos \varphi ^{\dag }\cos \theta ^{\dag }\\ -\sin ^2\varphi ^{\dag } \sin \theta ^{\dag }\cos \theta ^{\dag }\\ -\cos ^2\varphi ^{\dag }\cos ^2\theta ^{\dag } -\sin ^2\theta ^{\dag } \end{pmatrix}, \end{aligned} \end{aligned}$$

but more importantly, we remark that (A34) does not make sense at the North (and South) Pole, while (A43) does (and vice-versa (A34) is correct at the Equator, while(A43) is not defined there).

Finally, although we have not make use of this in the analysis of our interest, we remark that, since $\mathbf {\Omega }'\times (\mathbf {\Omega }'\times {\textbf{r}}')=\frac{1}{2}\nabla '(\Omega '^2\ell '^2)=:\nabla '\Phi '$, it is common to write the pressure as

A47 $$\begin{aligned} P'=p'-\rho ' g' \frac{R'^2 }{r'}+\rho '\Phi ', \end{aligned}$$

where $\ell '$ is the distance of the point from the axis or rotation ${\textbf{e}}_{3}$, and $\Phi '=-\frac{1}{2} r'^2\Omega '^2 (\cos ^2\varphi ^{\dag }\cos ^2\theta ^{\dag } + \sin ^2\theta ^{\dag })$.

The momentum equations with the re-defined pressure therefore become

A48 $$\begin{aligned} \begin{aligned}&\rho ' \frac{D}{D t'}\begin{pmatrix} u'_{\dag } \\ v'_{\dag }\\ w'_{\dag }\\ \end{pmatrix}+\frac{\rho '}{r'}\begin{pmatrix}-u'_{\dag }v'_{\dag }\tan \theta ^{\dag } + u'_{\dag }w'_{\dag }\\ u'^2_{\dag }\tan \theta ^{\dag }+v'_{\dag }w'_{\dag }\\ -u'^2_{\dag }-v'^2_{\dag } \end{pmatrix}\\&\quad + 2\rho '\Omega '\begin{pmatrix} -v'_{\dag }\sin \varphi ^{\dag }\cos \theta ^{\dag }-w'_{\dag }\sin \varphi ^{\dag }\sin \theta ^{\dag }\\ u'_{\dag }\sin \varphi ^{\dag }\cos \theta ^{\dag }-w'_{\dag }\cos \varphi ^{\dag }\\ u'_{\dag } \sin \varphi ^{\dag }\sin \theta ^{\dag } +v'_{\dag } \cos \varphi ^{\dag } \end{pmatrix}=\\&=-\nabla 'P'-\nabla '\rho '\left( \Phi '+g'\frac{R'^2}{ r'}\right) +\Delta '_{\mu } \begin{pmatrix} u'_{\dag }\\ v'_{\dag }\\ w'_{\dag } \end{pmatrix}-\frac{1}{3}\begin{pmatrix} \frac{1}{r'\cos \theta ^{\dag }}\frac{\partial }{\partial \varphi ^{\dag }}\left( \frac{\mu '_H}{\rho '} \frac{D\rho '}{D t'}\right) \\ \frac{1}{r'}\frac{\partial }{\partial \theta ^{\dag }}\left( \frac{\mu '_H}{\rho '} \frac{D\rho '}{D t'}\right) \\ \frac{\partial }{\partial r'}\left( \frac{\mu '_V}{\rho '} \frac{D\rho '}{D t'}\right) \end{pmatrix}\\&\quad -\frac{1}{r'^2\cos ^2\theta ^{\dag }}\begin{pmatrix} \mu '_H u'_{\dag }\\ \mu '_H v'_{\dag }\\ 2\mu '_V(w'_{\dag }\cos ^2\theta ^{\dag }-v'_{\dag }\sin \theta ^{\dag }\cos \theta ^{\dag }) \end{pmatrix}\\&\quad + \frac{2\mu '_H}{r'^2}\frac{\partial }{\partial \theta ^{\dag }}\begin{pmatrix} 0\\ w'_{\dag }\\ -v'_{\dag } \end{pmatrix}+\frac{2\mu '_H}{r'^2\cos \theta ^{\dag }}\frac{\partial }{\partial \varphi ^{\dag }}\begin{pmatrix} w'_{\dag }-v'_{\dag }\tan \theta ^{\dag }\\ u'_{\dag }\tan \theta ^{\dag }\\ -u'_{\dag } \end{pmatrix}\\&\quad +\frac{d \mu '_V}{dr'}r' \begin{pmatrix} \frac{\partial }{\partial r'}\left( \frac{u'_{\dag }}{r'}\right) \\ \frac{\partial }{\partial r'}\left( \frac{v'_{\dag }}{r'}\right) \\ {0} \end{pmatrix}+\frac{d \mu '_H}{d r'} \begin{pmatrix} \frac{1}{r'\cos \theta }\frac{\partial {w'_{\dag }}}{\partial \varphi ^{\dag }}\\ \frac{1}{r'} \frac{\partial {w'_{\dag }}}{\partial \theta ^{\dag }}\\ {0} \end{pmatrix}+ \frac{d \mu '_V}{dr'} \begin{pmatrix} 0\\ 0\\ 2 \frac{\partial w'_{\dag }}{\partial r'} \end{pmatrix}\\&\quad +\frac{d \mu '_V}{dr'}\begin{pmatrix} 0\\ 0\\ \frac{1}{r' \cos \theta ^{\dag }} \frac{\partial u'_{\dag }}{\partial \varphi ^{\dag }} + \frac{1}{r' \cos \theta ^{\dag }} \frac{\partial }{\partial \theta ^{\dag }} \left( v'_{\dag } \cos \theta ^{\dag } \right) + \frac{1}{r'^2} \frac{\partial }{\partial r'} \left( r'^2 w'_{\dag } \right) \end{pmatrix}. \end{aligned} \end{aligned}$$

This approach is particularly useful when considering constant density, in which case the term $\nabla '\rho '\left( \Phi '+g'\frac{R'^2}{ r'}\right)$ is zero.

## Footnotes

1 This runoff has been increasing as the hydrological cycle accelerates due to global warming [21]. The increase in runoff is already measurable, with a reported $9.8\%$ rise over the 30-year period from 1977 to 2007 [22].

2 We call “Ekman-type” spiral (or flow or current) a velocity vector of the form $e^{(1+i)sz}$ where $s\in {\mathbb {R}}^+\setminus \{\lambda \}=\{{\tilde{s}}\in {\mathbb {R}}\,:\, {\tilde{s}}>0\}\setminus \{\lambda \}$, while we call Ekman spiral (or flow or current) velocity vector of the form $e^{(1+i)\lambda z}$, where $\lambda =\sqrt{\frac{f}{2 A_V}}$.
